# High-intensity interval versus moderate-intensity continuous freestyle swimming in obese college students: A randomized controlled study protocol examining glycolipid and vascular endothelial responses

**DOI:** 10.1371/journal.pone.0360473

**Published:** 2026-10-30

**Authors:** Mei Yang, Jingjing Wang, Xiaoyun Su, Huan Xiang, Yuting Hou

**Affiliations:** 1 Department of Physical Education, Shanxi University, Taiyuan, Shanxi, China; 2 Faculty of Health and Wellness, City University of Macau, Macau, China; 3 Department of Nursing, Shanxi Medical College, Fenyang, Shanxi, China; Shiraz University, IRAN, ISLAMIC REPUBLIC OF

## Abstract

**Background:**

Obesity among college students may be accompanied by early metabolic and endothelial abnormalities before overt cardiometabolic disease develops. Freestyle swimming combines whole-body energy expenditure with low weight-bearing load. This randomized controlled trial will compare high-intensity interval training (HIIT) and moderate-intensity continuous training (MICT) freestyle swimming and examine the relationships between changes in glycolipid markers and vascular endothelial markers.

**Methods:**

A total of 108 obese college students will be randomized in a 1:1:1 ratio to HIIT, MICT, or a usual-activity control group (36 participants per group). The exercise groups will complete supervised 30-min freestyle swimming sessions three times weekly for 6 months. Total cholesterol (TC) at 6 months, adjusted for baseline TC, will be the single primary outcome. Secondary glycolipid, endothelial, anthropometric, adherence, and safety outcomes will be assessed at baseline and at 2, 4, and 6 months. The primary comparison will use analysis of covariance under the intention-to-treat principle. Longitudinal mixed-effects models and multiple imputation sensitivity analyses will characterize outcome trajectories. Multivariable linear regression will examine the relationships between changes in individual glycolipid markers and changes in ET-1 and NO.

**Discussion:**

This trial will clarify whether HIIT and MICT freestyle swimming differ in their effects on TC and other glycolipid and endothelial outcomes in obese college students and whether improvements in glycolipid markers are accompanied by changes in ET-1 and NO. The findings may inform safe and more precise exercise prescriptions for young adults with obesity.

**Clinical trial registration:**

The trial was prospectively registered in the Chinese Clinical Trial Registry (ChiCTR2600121931) on April 7, 2026.

## Introduction

Obesity is a chronic metabolic condition characterized by excessive body-fat accumulation and is closely associated with cardiovascular disease, type 2 diabetes, and metabolic syndrome [1]. National surveillance data from 291,276 Chinese college students aged 19–22 years showed that the prevalence of overweight and obesity increased to 23.4% and 11.4%, respectively, by 2019 [2]. Because college students are transitioning into adulthood, obesity-related metabolic abnormalities may still be subclinical even as future cardiometabolic risk accumulates. This population therefore represents an important target for early intervention [3].

Young adults with obesity are metabolically heterogeneous. Some meet criteria for metabolically healthy obesity (MHO), whereas others have metabolically unhealthy obesity (MUO) characterized by abnormalities in glycemia, lipids, or blood pressure [4,5]. Even when fasting glucose and lipid concentrations remain within conventional reference ranges, insulin sensitivity and metabolic flexibility may already be impaired. This high-normal or subclinical state may precede clinically apparent cardiometabolic disease [4,6].

In the context of declining metabolic regulation, early changes in vascular endothelial function are also relevant. The vascular endothelium helps maintain vasodilation, blood-flow regulation, and vascular homeostasis [5]. Under normal conditions, insulin stimulates endothelial nitric oxide synthase and nitric oxide (NO) production [7,8]. In obesity, impaired insulin signaling may reduce this vasodilatory response even before overt diabetes develops, contributing to subclinical endothelial dysfunction and future cardiovascular risk [9,10].

Exercise is an effective non-pharmacological strategy for obesity and related metabolic risk [11,12]. Aerobic exercise increases energy expenditure, improves insulin sensitivity, and regulates lipid metabolism [11]. Swimming recruits multiple muscle groups while limiting weight-bearing joint load, which may improve tolerability in people with higher body weight. A meta-analysis of randomized trials found that regular aquatic endurance exercise can improve lipid and lipoprotein profiles [13].

Most exercise studies in populations with obesity have evaluated overall metabolic health, and few have directly compared swimming intensities while assessing both glycolipid regulation and vascular endothelial function [14,15]. It therefore remains unclear whether HIIT and MICT produce different time courses or magnitudes of endothelial adaptation in obese college students whose metabolic indicators may still lie within conventional reference ranges.

Exercise can influence both metabolic regulation and endothelial function, and the magnitude of these responses may be related [7–10,16]. We will therefore examine whether changes from baseline in individual glycolipid markers are associated with concurrent changes in ET-1 and NO. The analysis will retain the original scale and clinical interpretation of each marker.

Accordingly, this study will compare 6 months of HIIT and MICT freestyle swimming in obese college students. We hypothesize that both training modes will improve TC and secondary metabolic and endothelial outcomes relative to control, that HIIT will produce earlier or larger changes in insulin-related and ET-1/NO outcomes, and that changes in glycolipid markers will be associated with concurrent changes in ET-1 and NO.

### Participant timeline

Participant recruitment is planned to start on April 10, 2026, and the trial is expected to be completed by November 2026. Eligible participants will be screened, enrolled, randomized, and assessed according to the predefined schedule. Baseline measurements will precede allocation, and follow-up assessments will occur at 2, 4, and 6 months. The SPIRIT schedule of enrolment, interventions, and assessments is shown in Fig 1.

**Fig 1:**
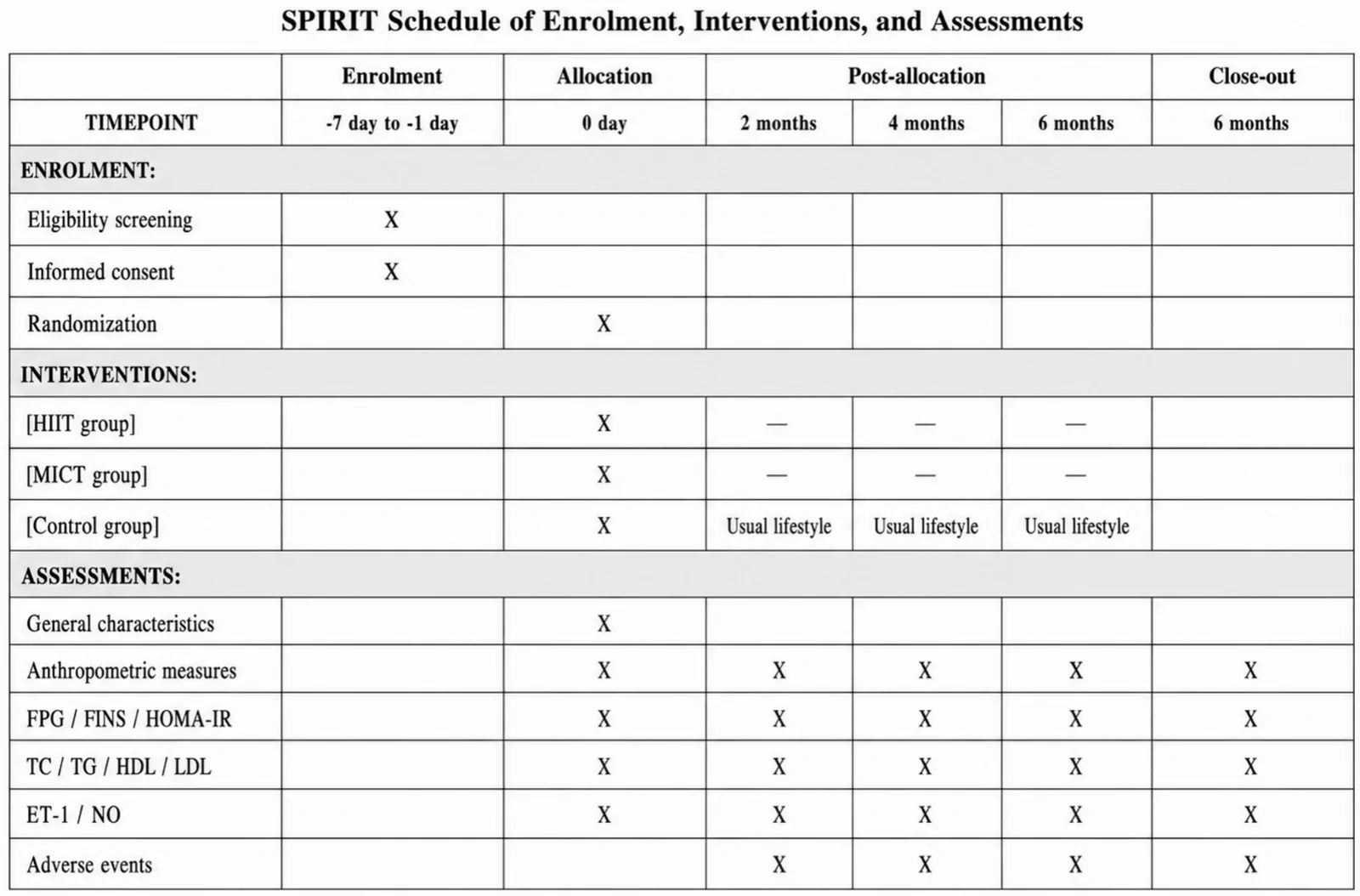
SPIRIT 2025 schedule of enrolment, interventions, and assessments.

## Materials and methods

### Study design and ethics

This protocol describes a three-arm, parallel-group, assessor-blinded randomized controlled trial and is reported in accordance with the SPIRIT 2025 statement [17]. The Scientific Research Ethics Review Committee of Shanxi University approved the protocol (SXULL2026081), and the trial was prospectively registered in the Chinese Clinical Trial Registry (ChiCTR2600121931). Before enrollment, each participant will receive an explanation of the study aims, procedures, potential benefits, and risks and will provide written informed consent. The trial will be conducted in accordance with the Declaration of Helsinki. The prospective participant flow is shown in Fig 2.

**Fig 2:**
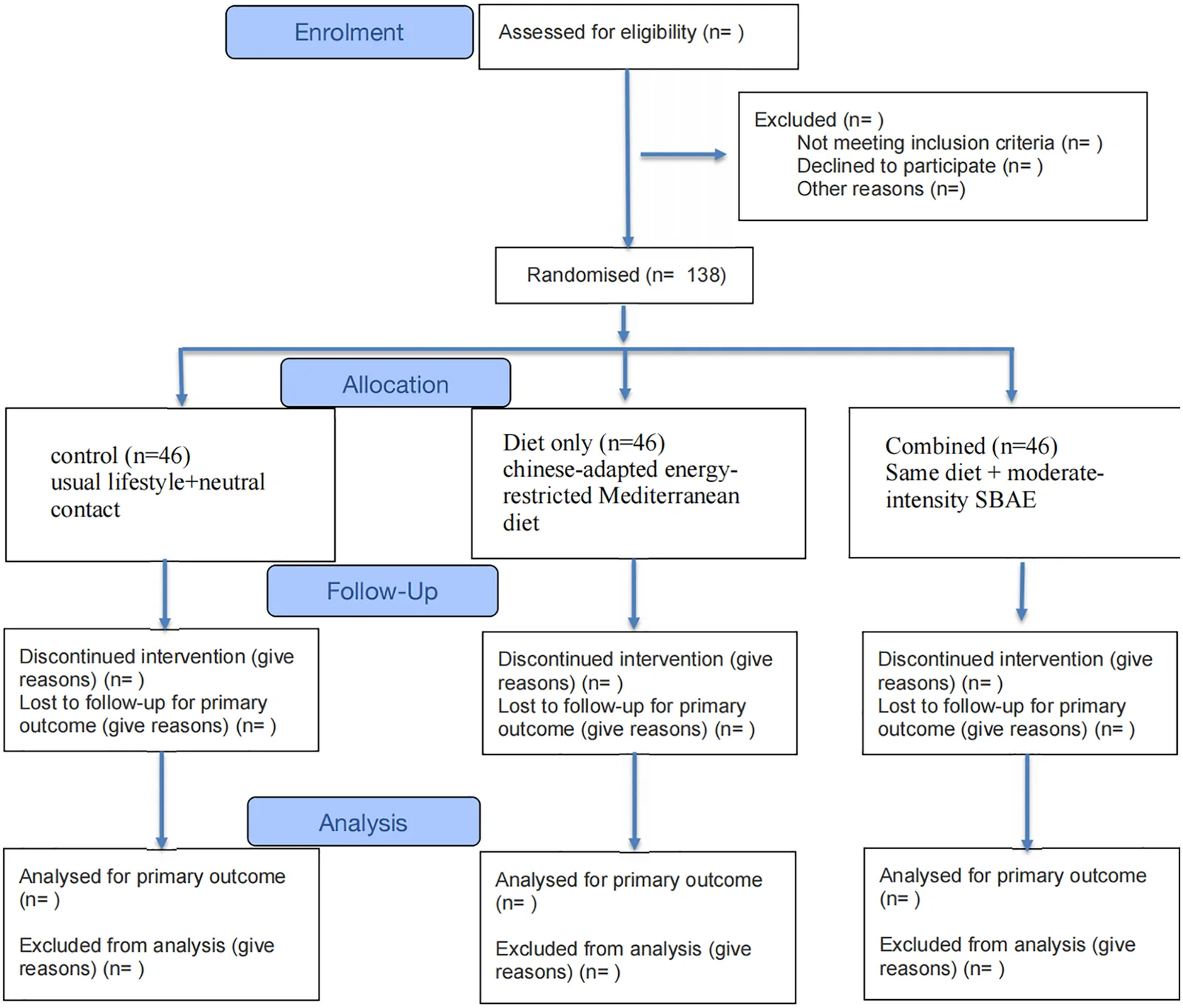
Prospective SPIRIT participant flow diagram. The target randomized sample is 108 participants, with 36 allocated to each group; actual screening, follow-up, and analysis counts will be reported after trial completion.

### Participants and sample size

Participants will be recruited from Shanxi University through campus advertisements, online notices, and voluntary enrolment. All potential participants will undergo a standardized screening procedure before formal inclusion to determine their eligibility for the study. The target population will consist of full-time college students aged 18–22 years who are non-sports majors and have not received systematic athletic training.

Eligible participants must meet all of the following criteria: (1) age 18–22 years; (2) body mass index (BMI) >28 kg/m^2^; (3) body-fat percentage >20% for males or >28% for females, measured by dual-energy X-ray absorptiometry (DEXA; Fig 3); (4) stable body weight during the preceding 3 months (change no greater than ±2 kg); (5) no diagnosed major chronic disease that would preclude exercise; (6) no history of systematic athletic training; and (7) willingness and ability to complete the intervention and assessment schedule and provide written informed consent.

**Fig 3:**
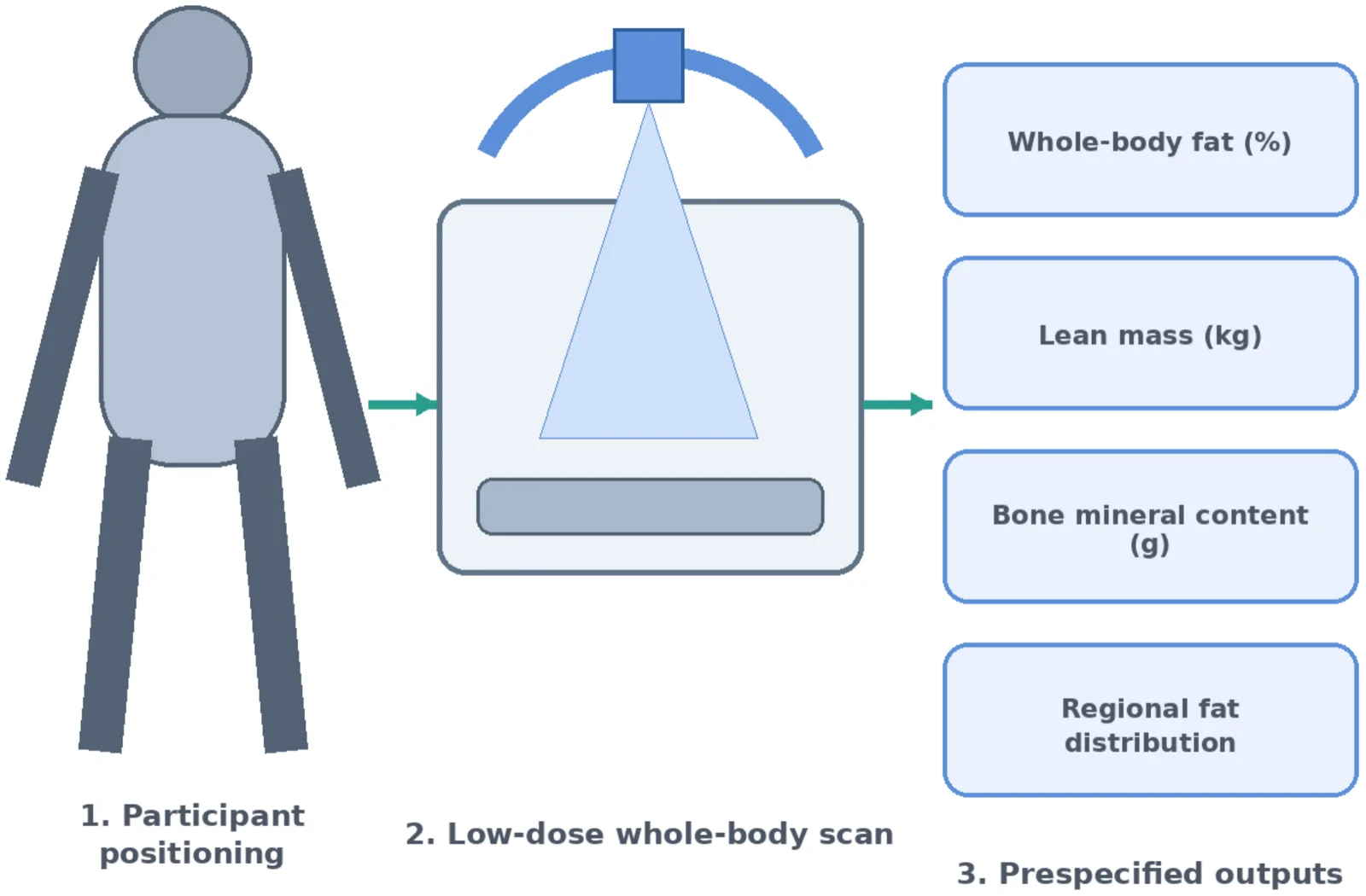
De-identified schematic of DEXA body-composition assessment and prespecified outputs. No participant identifiers or individual clinical records are shown.

Exclusion criteria are: (1) cardiovascular, infectious, or other disease that makes exercise unsafe; (2) any contraindication to exercise testing or swimming; (3) a clear family history of hereditary obesity; (4) a chronic condition or current medication expected to materially influence glycolipid metabolism or vascular function; (5) current smoking, alcohol abuse, pregnancy, or another factor that could compromise safety or interpretation; or (6) inability to complete blood collection, physical assessment, or exercise testing.

Before randomization, candidates will complete a structured medical history, medication review, resting blood-pressure and heart-rate assessment, physical examination, exercise-readiness screening, baseline fasting biochemistry, and a standardized swimming-ability evaluation. A study physician will review abnormal findings and determine exercise eligibility. Baseline metabolic phenotype will be classified for descriptive and exploratory analyses using prespecified MHO criteria: triglycerides ≤1.7 mmol/L, HDL-C > 1.0 mmol/L in males or >1.3 mmol/L in females, blood pressure ≤130/85 mmHg, fasting glucose ≤6.1 mmol/L, no relevant drug treatment, and no manifest cardiovascular disease [22]. Participants not meeting all criteria will be classified as MUO. Both phenotypes will remain eligible because the trial addresses obese college students across the metabolic-risk spectrum; phenotype will be included as a prespecified covariate and exploratory effect modifier.

TC concentration at 6 months, adjusted for baseline TC, is the single primary outcome and the basis of the sample-size calculation. Published exercise-intervention data were used instead of an unpublished pilot dataset. In a 6-week aquatic trial, Tang et al. reported that TC decreased from 5.36 ± 0.84 to 4.96 ± 1.00 mmol/L after aquatic HIIT and from 5.06 ± 0.81 to 5.03 ± 0.40 mmol/L after aquatic MICT; Saeidi et al. reported an effect size of 0.90 for the group-by-time comparison of TC in adults with obesity [23,24]. Because the source populations, interventions, and follow-up periods differ from the present trial, a smaller, conservative moderate omnibus effect was prespecified for planning (Cohen’s f = 0.325; equivalent eta-squared = f-squared/(1 + f-squared) = 0.096). An a priori calculation with G*Power version 3.1.9.7 [25] used F tests, ANOVA: fixed effects, omnibus, one-way, three groups, alpha = 0.05, and power = 0.80. The minimum total sample was 95 and was rounded to 96 to permit equal allocation (32 per group). This one-way omnibus calculation provides a conservative approximation for the planned baseline-adjusted primary ANCOVA. Allowing 10% attrition gives 96/(1–0.10) = 106.7; this was rounded up to 108 to preserve 1:1:1 allocation (36 per group).

### Randomization and blinding

An independent statistician who is not involved in recruitment, intervention delivery, or outcome assessment will generate a computer-based sequence with concealed variable block sizes. Allocation will be centralized and stratified by sex and baseline BMI category (<30 or ≥30 kg/m^2^), followed by 1:1:1 assignment to HIIT, MICT, or control. Sex and BMI were selected because they are expected to influence body composition, metabolic risk, and exercise response; height and weight will be recorded but will not be used as separate strata because they are components of BMI. Group assignment cannot be masked from participants or exercise supervisors, but outcome assessors and the statistician conducting the primary analysis will remain blinded until the primary analysis is finalized.

### Intervention protocol

Before the formal trial, participants allocated to either exercise arm will complete preliminary freestyle instruction. Only participants able to tread water for at least 30 s and swim continuously for at least 200 m will enter the intervention phase. Both prescriptions follow the FITT-VP principle and use the same session duration: 5 min of warm-up, 20 min of primary training, and 5 min of cool-down, three times weekly for 6 months. Heart rate will be recorded throughout each session with a Polar Verity Sense optical sensor to verify exposure to the target intensity zones.

The HIIT group will perform repeated 30-s freestyle intervals at 80–85% of estimated maximal heart rate, interspersed with 30-s active recovery at 50–60% of maximal heart rate. During weeks 1–2, the number of intervals will progress from 16 to 20. The MICT group will perform moderate-intensity freestyle swimming at 60–70% of maximal heart rate, with light recovery swimming at ≤60% of maximal heart rate. Training volume will be titrated during the first 2 weeks to improve tolerance. The control group will maintain usual daily activities and will receive no structured exercise training (Table 1).

**Table 1:** Intervention protocols based on the FITT-VP principle.

FITT-VP component	HIIT group	MICT group	Control group
Frequency	3 sessions/week	3 sessions/week	No structured exercise intervention
Intensity	Sprint intervals: 80–85% HRmaxActive recovery: 50–60% HRmax	Main swimming sets: 60–70% HRmaxLight recovery: ≤ 60% HRmax	Daily activities only
Time	30 min/session	30 min/session	–
Type	Freestyle swimming; interval-based high-intensity training	Freestyle swimming; moderate-intensity continuous training	–
Volume	5 min warm-up + 20 min (30 s/30 s x 20 cycles) + 5 min cool-down	5 min warm-up + 20 min segmented continuous swimming + 5 min cool-down	–
Progression	Weeks 1–2: 16–20 intervals; progression by increasing time in target HR zone	Weeks 1–2: 4–5 sets; progression by improved tolerance to continuous workload	–
Intervention duration	6 months	6 months	6 months

HRmax, maximal heart rate; HIIT, high-intensity interval training; MICT, moderate-intensity continuous training.

### Outcome measures

#### Primary outcome.

The single primary outcome is TC concentration at 6 months. The primary estimand is the mean between-group difference in 6-month TC under assignment to HIIT, MICT, or control, regardless of adherence or post-randomization deviations, with adjustment for baseline TC and the randomization stratification factors.

#### Secondary outcomes.

Secondary glycolipid outcomes are TC at 2 and 4 months and fasting plasma glucose (FPG), fasting insulin (FINS), homeostatic model assessment for insulin resistance (HOMA-IR), triglycerides (TG), high-density lipoprotein cholesterol (HDL-C), and low-density lipoprotein cholesterol (LDL-C) at 2, 4, and 6 months. Anthropometric secondary outcomes include body weight, BMI, body-fat percentage, waist-to-hip ratio, and chest, waist, arm, and leg circumferences at the same time points.

#### Vascular endothelial outcomes and regression analyses.

Serum endothelin-1 (ET-1) and nitric oxide (NO) will be assessed at baseline and at 2, 4, and 6 months as secondary markers of vasoconstrictive and vasodilatory endothelial adaptation, respectively. Relationships between metabolic and endothelial responses will be examined using changes from baseline in the individual markers, with ET-1 and NO modeled separately as dependent variables.

#### Baseline characteristics.

Age, sex, height, weight, BMI, body-fat percentage, waist-to-hip ratio, chest circumference, waist circumference, arm circumference, leg circumference, blood pressure, current medication, and metabolic phenotype will be recorded before randomization. These data will describe the sample, support prespecified adjustment, and characterize baseline balance; they will not determine whether baseline covariates enter the primary model.

#### Blood collection and laboratory assays.

Participants will avoid vigorous exercise and alcohol for 24 h and will complete a 10–12 h overnight fast before sampling. Venous blood will be collected between 07:00 and 09:00 at each assessment. Fluoride-oxalate plasma will be used for FPG, and serum-separator tubes will be used for insulin, lipids, ET-1, and NO. Serum will clot for 30 min and will then be centrifuged at 1,500 x g for 10 min at 4°C within 60 min of collection. FPG will be measured by a hexokinase method and TC, TG, HDL-C, and LDL-C by enzymatic colorimetric assays. FINS and ET-1 will be measured by sandwich enzyme-linked immunosorbent assay, and NO as total nitrate/nitrite by a Griess-based colorimetric assay, using commercially available kits manufactured by Elabscience Biotechnology Co., Ltd. (Wuhan, China) in accordance with the manufacturer’s instructions. HOMA-IR will be calculated as fasting glucose (mmol/L) x fasting insulin (microU/mL)/22.5. Serum aliquots will be stored at −80°C, participant samples will be analyzed in the same batch where feasible, and duplicate measurements and internal quality-control materials will be used. Coefficients of variation greater than 10% will trigger repeat testing when sufficient sample remains. The catalog number, production lot number, detection range, and intra- and inter-assay coefficients of variation of the actual Elabscience kits used will be recorded during the trial and reported in the final results article.

### Safety outcomes

Safety outcomes will include all adverse events during the intervention, including dizziness, chest tightness, palpitations, excessive fatigue, muscle soreness or cramps, joint or soft-tissue discomfort, slipping, and other exercise-related symptoms. For each event, severity, timing, relatedness, management, and outcome will be recorded. Heart rate and symptoms will be monitored during every session, and training will be paused or terminated when prespecified safety criteria are met. Serious adverse events will be reported promptly to the principal investigator and ethics committee.

### Quality control and safety monitoring

To reduce dietary confounding, participants will be asked to maintain their habitual diet and avoid deliberate caloric restriction or overeating. Intake will be logged with the Boohee Health application and reviewed periodically. Attendance, time in the prescribed heart-rate zone, session completion, medication changes, dietary changes, and non-study exercise will be recorded. No participant or observation will be removed from the intention-to-treat analysis because of poor adherence or a post-randomization lifestyle change. A per-protocol sensitivity analysis will be limited to participants completing at least 90% of prescribed sessions without a major prohibited co-intervention.

All swimming sessions will be supervised in a facility equipped for water safety. Heart rate and symptoms will be monitored in real time, and a session will be paused or terminated if a participant develops dizziness, chest tightness, palpitations, unusual dyspnea, or excessive fatigue. Adverse events and protocol deviations will be reviewed at predefined intervals throughout follow-up.

### Statistical analysis

Analyses will be conducted with SPSS version 25.0 and R. A detailed, outcome-neutral statistical analysis plan will be finalized before group codes are released to the primary analyst. Continuous variables will be summarized as mean (standard deviation) or median (interquartile range), as appropriate, and categorical variables as number (percentage). Distributional assumptions will be evaluated with residual plots and Q-Q plots rather than by significance tests alone.

Baseline characteristics will be described by randomized group using standardized mean differences and confidence intervals. Baseline hypothesis tests will not be used to select covariates or determine the primary model. Baseline TC and the randomization stratification factors (sex and BMI category) will be included in the primary model regardless of observed balance.

The primary analysis will follow the intention-to-treat principle and will compare 6-month TC among the three groups using analysis of covariance (ANCOVA), with 6-month TC as the dependent variable and randomized group, baseline TC, sex, and BMI category as prespecified predictors. The omnibus group test will be evaluated at a two-sided alpha of 0.05. If supported, the three prespecified pairwise contrasts (HIIT vs control, MICT vs control, and HIIT vs MICT) will be reported as adjusted mean differences with 95% confidence intervals and Holm-adjusted P values.

Secondary repeated outcomes will be analyzed with linear mixed-effects models containing fixed effects for group, time as a categorical variable, group x time interaction, the baseline value of the outcome, sex, and BMI category, plus a participant-specific random intercept. Estimated marginal means, adjusted between-group differences, and 95% confidence intervals will be reported at each time point. Model residuals will be examined; transformations or generalized mixed models will be used when distributional assumptions are materially violated. Secondary biomarker results will be interpreted with false-discovery-rate control within prespecified outcome families.

All randomized participants will remain in the primary analysis. Reasons for withdrawal and the timing and pattern of missingness will be summarized by group. Mixed-effects models will use maximum likelihood under a missing-at-random assumption. For the primary 6-month ANCOVA, missing outcome or covariate values will be addressed by multiple imputation using chained equations that include group, baseline and prior outcome values, stratification variables, adherence, and predictors of missingness. Estimates will be pooled with Rubin’s rules. Complete-case and per-protocol analyses, and a sensitivity analysis under departures from the missing-at-random assumption, will be reported to assess robustness.

Additional multivariable linear regression analyses will examine the relationships between metabolic and endothelial responses at 2, 4, and 6 months. At each time point, change from baseline in ET-1 and change from baseline in NO will be analyzed as separate dependent variables. Changes from baseline in TC, TG, HDL-C, LDL-C, FPG, FINS, and HOMA-IR will be entered separately as the metabolic predictor in prespecified models to reduce multicollinearity. Each model will adjust for randomized group, the baseline value of the endothelial outcome, sex, and BMI category. Standardized regression coefficients with 95% confidence intervals and P values will be reported. Linearity, homoscedasticity, residual distribution, and influential observations will be assessed, and false-discovery-rate adjustment will be applied across the metabolic predictors for each endothelial outcome at each time point.

All tests will be two-sided. The primary omnibus test will use P < 0.05; adjusted pairwise and secondary-family procedures are described above. Effect estimates with 95% confidence intervals will be emphasized. Regression results will be reported as adjusted standardized coefficients with 95% confidence intervals. No formal efficacy claim will be based solely on a subgroup or per-protocol analysis.

### Use of artificial intelligence

OpenAI ChatGPT was used during manuscript revision to support language editing. The authors will independently verify all sources and approve every methodological and statistical decision and will retain full responsibility for the final article. No AI tool was used to generate, fabricate, or analyze study data.

## Discussion

Obesity in college students represents an early and potentially reversible stage of cardiometabolic risk. Although many young obese individuals have not yet developed overt metabolic disease, subtle impairments in glycolipid regulation and vascular endothelial function may already be present. Early intervention at this stage is therefore of particular importance, because it may help prevent the transition from a high-normal metabolic state to clinically significant cardiovascular and metabolic abnormalities. In this context, identifying an effective and feasible exercise strategy for obese college students is of both scientific and practical value.

Freestyle swimming is suitable for this population because it combines whole-body muscular recruitment with relatively low weight-bearing joint load [18]. Both interventions may improve glucose uptake, insulin sensitivity, and lipid oxidation through increased energy turnover and skeletal-muscle activation. Repeated blood-flow shear stress may also increase NO bioavailability and reduce vasoconstrictor activity(Fig 4).HIIT may provide a larger intermittent metabolic and hemodynamic stimulus, whereas MICT may produce a more gradual adaptation during the 6-month intervention [19–21]. The protocol does not include post-intervention follow-up, so persistence after exercise cessation cannot be evaluated.

**Fig 4:**
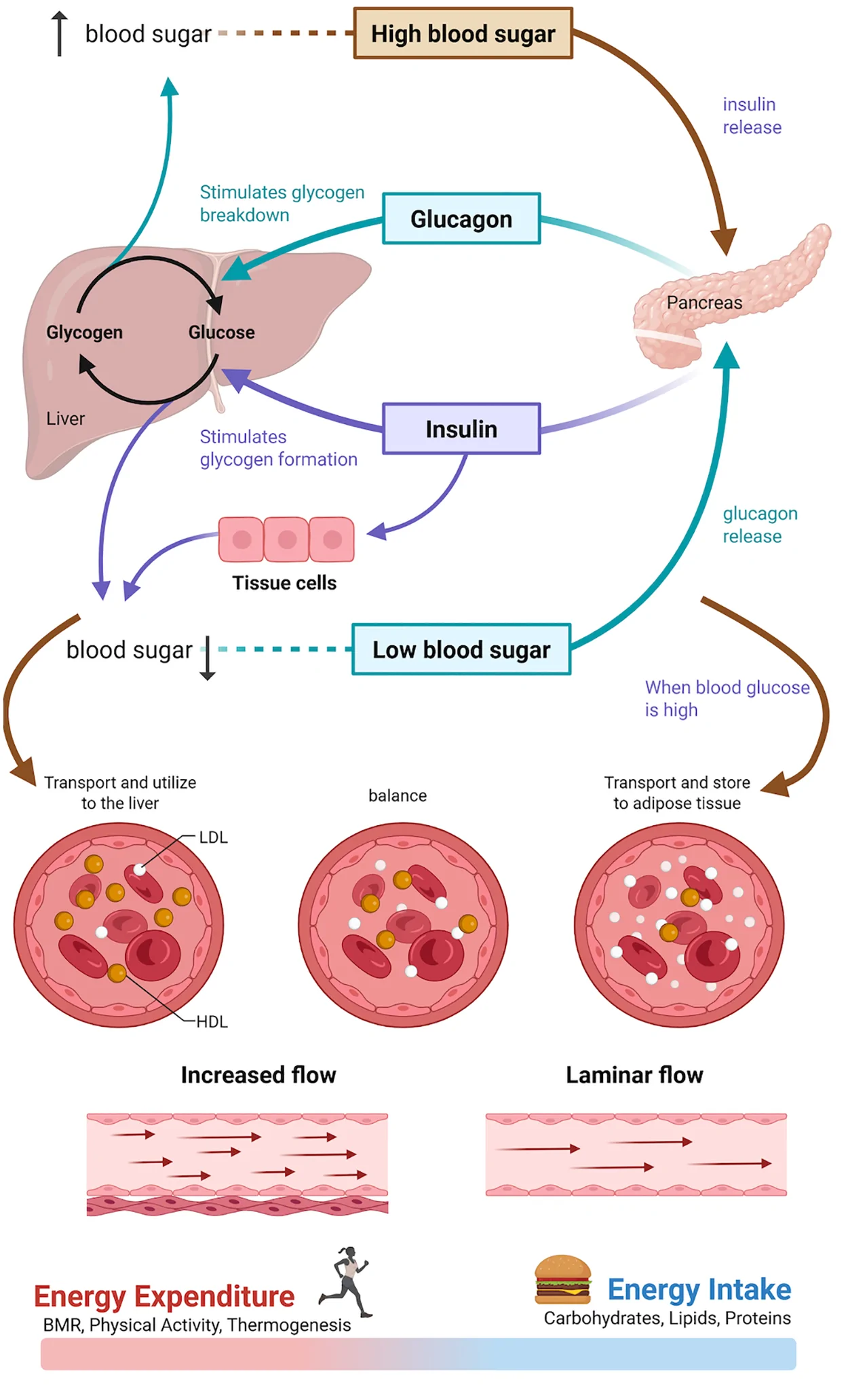
Conceptual framework linking glucose regulation, lipid transport, vascular blood-flow patterns, and whole-body energy balance. High blood glucose stimulates pancreatic insulin release, supporting cellular glucose uptake and hepatic glycogen formation; low blood glucose stimulates glucagon release and hepatic glycogen breakdown. The middle panels illustrate lipid transport to the liver or storage in adipose tissue according to metabolic state, together with exercise-related increases in blood flow and laminar shear. The lower panel represents the balance between energy expenditure and energy intake. These physiological relationships provide the rationale for assessing glycolipid and endothelial outcomes in the present trial.Illustration created in BioRender. Yang, M. (2026) https://BioRender.com/fszg4u4.

A key strength of this protocol is the comparison of two training intensities within the same exercise modality, which reduces confounding by exercise type. Repeated assessments at baseline and at 2, 4, and 6 months will characterize the timing of TC, other metabolic outcomes, ET-1, and NO changes. The regression analyses will show whether changes in specific glycolipid markers are related to concurrent changes in ET-1 and NO after adjustment for randomized group and baseline factors.

The analytical hierarchy also limits undisclosed flexibility. ANCOVA with baseline adjustment is prespecified for the single primary outcome, mixed-effects models address repeated secondary outcomes, and intention-to-treat inference is separated from per-protocol sensitivity analysis. Explicit missing-data procedures will preserve randomized comparisons and quantify the robustness of conclusions. This hierarchy replaces conditional baseline adjustment and avoids treating multiple biomarkers as equivalently powered primary outcomes.

The expected value of this study is threefold. First, it may provide evidence for a more precise exercise prescription for obese college students by clarifying whether HIIT offers additional advantages over MICT under safe and supervised conditions. Second, it will describe the metabolic and endothelial responses during the 6-month intervention and evaluate whether the magnitudes of change in these two outcome domains are related. Third, because the target population is young and at a relatively early stage of metabolic dysfunction, the findings may have particular relevance for early prevention.

Several limitations should be acknowledged. The single-center university sample may limit generalizability. MHO and MUO participants may respond differently; phenotype will therefore be classified at baseline and examined as a covariate and exploratory effect modifier, but the trial is not powered for phenotype-specific effects [22]. The sample size is based on TC, so the secondary endothelial biomarkers and additional regression analyses may have limited precision. ET-1 and NO are circulating surrogate markers and do not provide a direct vascular imaging measure. Diet and non-study activity are monitored partly by self-report, leaving residual confounding possible. Finally, the last assessment coincides with the end of the 6-month intervention, so the durability of adaptation after detraining cannot be determined.

### Confidentiality

All participant information collected during the study will be kept strictly confidential. Personal identifiers will be removed from the research dataset and replaced with unique study codes before data entry and analysis. Only authorized members of the research team will have access to identifiable information, and all study records, including informed consent forms, case report forms, and laboratory results, will be securely stored in locked files or password-protected electronic databases. The results of this study will be reported in aggregate form only, and no individual participant will be identifiable in any publication or public report. All data will be managed and retained in accordance with institutional requirements for research involving human participants.

### Trial status

This is protocol version 1.0. Participant recruitment is scheduled to begin on April 10, 2026, and is expected to be completed by November 2026.

## Supporting information

S1 File SPIRIT 2025 checklist filled. (DOCX)

S2 File Original protocol. (PDF)
