# Supplementary material for: High-intensity interval versus moderate-intensity continuous freestyle swimming in obese college students: A randomized controlled study protocol examining glycolipid and vascular endothelial responses
Source: PLoS One. 2026 Oct 30;21(10):e0360473. doi: 10.1371/journal.pone.0360473 (PMC13722204; doi:10.1371/journal.pone.0360473)
Supplement: Supplementary file 1 [file pone.0360473.s002.pdf]

Effects of Different Swimming Exercise Intensities on Glycolipid Metabolism and Vascular Endothelial Function in Obese College Students: An Intervention Study

(Version V1.0)

Part 1. Project Summary

This study systematically investigates the differential effects of a 6-month high-intensity interval swimming program versus a moderate-intensity continuous swimming program on glycolipid metabolism and vascular endothelial function in obese college students, and the associations between them. Using a randomized controlled trial design, 108 obese college students will be randomized in a 1:1:1 ratio to a high-intensity interval training (HIIT) group, a moderate-intensity continuous training (MICT) group, or a no-intervention control group (36 per group). Glycolipid metabolism markers, lipid profiles, and vascular endothelial biomarkers will be assessed at baseline and at 2, 4, and 6 months. The primary outcome will be analyzed using baseline-adjusted analysis of covariance; longitudinal outcomes will be analyzed with linear mixed-effects models; and multivariable linear regression will examine the relationship between changes in glycolipid markers and changes in vascular function. This study is expected to clarify the differential effects of swimming intensity on metabolic health and vascular function and their underlying mechanisms, providing high-level evidence for precise and efficient exercise interventions for obesity in college students.

Part 2. Background and Rationale

1. Problem statement and current evidence

Globally, obesity prevalence among college students has risen to 10%-20%, making this group an important early population for type 2 diabetes, atherosclerosis, and other metabolic diseases. In obesity, insulin resistance and dyslipidemia interact to jointly impair vascular endothelial function, which is an initiating event in cardiovascular events. Exercise is a first-line non-pharmacological intervention whose metabolic benefits are well established, yet the 'optimal exercise intensity' remains controversial.

Existing studies have focused mainly on land-based exercise such as running and cycling, with limited attention to swimming—a low-joint-load, whole-body exercise. Limited evidence indicates that swimming improves body composition and insulin sensitivity, but systematic, high-quality longitudinal research is lacking on the combined glycolipid benefits of different swimming intensities in obese college students and on the causal relationship with improved vascular endothelial function.

2. Theoretical basis and hypotheses

Theoretical basis:

1. Exercise intensity and metabolic adaptation theory: HIIT, through repeated near-maximal bouts, deeply activates the AMPK signaling pathway and mitochondrial biogenesis, and may be superior to MICT in improving insulin sensitivity and fat oxidation.

2. Blood-flow shear stress and endothelial function theory: increased blood-flow shear stress during exercise is a key physical stimulus that upregulates endothelial nitric oxide synthase, promotes NO release, and inhibits ET-1 production; different exercise intensities may produce different shear-stress patterns and thus different vascular effects.

3. Metabolism-vascular axis theory: improvements in glycolipid metabolism reduce systemic inflammation and oxidative stress, thereby reducing endothelial injury and forming a virtuous cycle between metabolic and vascular improvement.

Hypotheses:

1. Compared with control, both HIIT and MICT will significantly improve glycolipid metabolism and vascular endothelial function in obese college students.

2. HIIT will be superior to MICT in reducing fasting insulin, HOMA-IR, and TG and in raising HDL.

3. The magnitude of improvement in glycolipid markers will be significantly associated with changes in vascular endothelial markers.

3. Innovations

1. Focused population: specifically targets obese college students, a high-risk group underrepresented in intervention research.

2. Exercise modality specificity: focuses on swimming, providing evidence distinct from land-based exercise.

3. Mechanistic depth: for the first time in a swimming intervention, simultaneously and longitudinally monitors glucose metabolism, lipid metabolism, and vascular endothelial function, and analyzes their interrelationships.

4. Methodological rigor: uses baseline-adjusted analysis of covariance and linear mixed-effects models to estimate the net intervention effect while controlling for time and other confounders.

Part 3. Study Design

1. Overall design

A three-arm, randomized, controlled, prospective intervention study. The total duration is 8 months, comprising 1 month of baseline assessment, 6 months of intervention, and 1 month of data cleaning and analysis.

2. Technical route

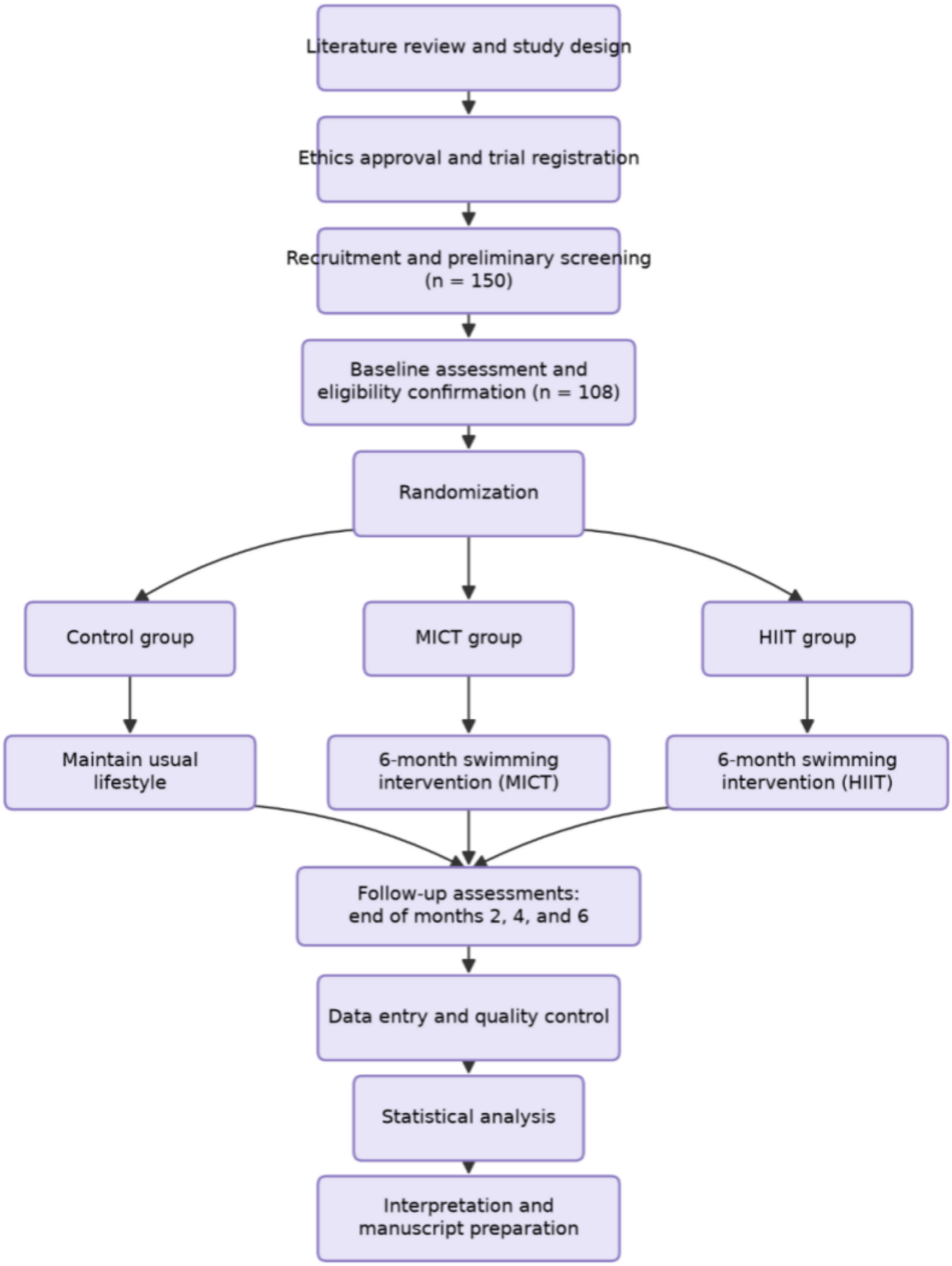

3. Participants

Sample size calculation: The single primary outcome is total cholesterol (TC) at 6 months, adjusted for baseline TC. The sample size was based on published aquatic-exercise data (group-by-time effects for TC reported by Tang et al. and Saeidi et al.), with a prespecified conservative

moderate omnibus effect (Cohen's f = 0.325; equivalent eta-squared = 0.096). Using G*Power 3.1.9.7 with an F test (one-way ANOVA, fixed effects, omnibus, three groups), two-sided alpha = 0.05, and power = 0.80, the minimum total sample was 95; this was rounded to 96 to allow equal allocation (32 per group). Allowing 10% attrition, 96/(1-0.10) = 106.7, rounded up to 108, i.e., randomized 1:1:1 to three groups with 36 participants per group. Recruitment channels: campus advertisements, online notices, and voluntary enrollment at Shanxi University.

Inclusion criteria (all must be met):

- Full-time undergraduate students (non-sports majors, no systematic athletic training), aged 18-22 years.

- Body mass index (BMI) > 28 kg/m^2.

- Body-fat percentage > 20% in males or > 28% in females, measured by dual-energy X-ray absorptiometry (DEXA).

- Stable body weight over the preceding 3 months (change no greater than ±2 kg), and no regular moderate-intensity exercise habit over the preceding 6 months.

- Provide written informed consent.

Exclusion criteria (any one):

- Cardiovascular, infectious, or other disease that makes exercise unsafe; or a clear family history of hereditary obesity.

- Chronic conditions or current medication expected to materially influence glycolipid metabolism or vascular function (e.g., antidiabetic drugs, statins, hormones).

- Any contraindication to exercise testing or swimming.

- Current smoking, alcohol abuse, pregnancy, or other factors that could compromise safety or interpretation.

- Planned participation in other weight-loss programs or surgery during the study; or inability to complete blood collection, physical assessment, or exercise testing.

4. Randomization and blinding

Randomization: An independent statistician not involved in recruitment, intervention delivery, or outcome assessment will generate a computer-based allocation sequence using concealed variable block sizes. Allocation will be centralized and stratified by sex and baseline BMI category (<30 or ≥30 kg/m^2), followed by 1:1:1 assignment to HIIT, MICT, or control.

Blinding: Because of the nature of the exercise intervention, participants and exercise supervisors cannot be blinded. Outcome assessors (responsible for measurement and blood collection) and the data analyst will be blinded to group assignment.

Part 4. Intervention Protocol and Assessment Methods

1. Intervention details

Setting: A standard university heated swimming pool (water temperature 28 ± 1°C).

Supervision: Before the formal trial, participants in both exercise arms will complete preliminary freestyle instruction; only those able to tread water for at least 30 seconds and swim continuously for at least 200 m will enter the intervention phase. Each group will be supervised by one coach holding a lifeguard certificate and sports-medicine training and one research assistant. Heart rate will be monitored and recorded throughout using a Polar Verity Sense optical heart-rate sensor to verify attainment of the target intensity zones.

HIIT protocol:

- Both groups use a unified session structure: 5 min warm-up, 20 min main training, 5 min cool-down, approximately 30 min per session, three times weekly for 6 months.

- HIIT main training: freestyle intervals of 30 s at 80-85% of estimated maximal heart rate (HRmax), interspersed with 30 s active recovery at 50-60% HRmax; the number of intervals will progress from 16 to 20 during weeks 1-2 to improve tolerance.

- Cool-down: 5 min low-intensity relaxation.

- Total HIIT session duration: approximately 30 min.

MICT protocol:

- MICT main training: continuous moderate-intensity freestyle swimming at 60-70% HRmax, with light recovery swimming at no more than 60% HRmax.

- Cool-down: 5 min low-intensity relaxation.

- Total MICT session duration: approximately 30 min.

Frequency and adherence: Both groups train three times weekly (alternating days). Adherence is defined as actual attendance / planned sessions ≥ 80%; participants missing more than 3 sessions will undergo a make-up program, and more than 5 absences will be considered dropout.

Control group: No structured exercise intervention; a health lecture will be offered every 2 months (content not involving exercise prescription) to maintain contact.

2. Outcomes and time points

All assessments will be performed after a 10-12 h overnight fast, between 07:00 and 09:00, under quiet conditions.

Time points: T0 (baseline), T1 (2 months), T2 (4 months), T3 (6 months).

Anthropometric measures:

- Height, weight, BMI.

- Body-fat percentage (DEXA).

- Waist circumference, hip circumference, waist-to-hip ratio.

Blood biochemistry:

Glucose metabolism: FPG (hexokinase method), FINS (sandwich ELISA), HOMA-IR (= fasting glucose mmol/L × fasting insulin μU/mL / 22.5).

Lipid metabolism: TC, TG, HDL-C, LDL-C (enzymatic colorimetric assay, automated biochemical analyzer).

Vascular endothelial function: serum NO (total nitrate/nitrite, Griess colorimetric assay) and plasma ET-1 (sandwich ELISA); commercial kits from Elabscience Biotechnology Co., Ltd. will be used, and repeat testing will be performed when intra-/inter-assay CV exceeds 10%.

Exercise capacity (T0 and T3 only): maximal oxygen uptake estimated using a pool-based indirect test.

3. Data management and quality control

Data collection: Dedicated case report forms with double independent entry into an electronic database (REDCap).

Blood handling: standardized collection, centrifugation, aliquoting, and storage at -80°C; each marker will be assayed in a single batch.

Personnel training: all research staff, nurses, and technicians will receive standardized SOP training.

Process monitoring: As a single-center, relatively low-risk exercise intervention, no formal independent data monitoring committee will be established; participant safety and trial conduct will be monitored by the principal investigator and intervention supervisors. Adverse events and protocol deviations will be reviewed regularly, and any serious safety concern will be documented and reported promptly to the university ethics committee.

Part 5. Statistical Analysis Plan

1. Descriptive statistics: continuous data as mean ± SD or median; categorical data as frequency (percentage).

2. Baseline balance: one-way ANOVA or chi-square tests across the three groups.

3. Primary outcome analysis (intention-to-treat): 6-month TC as the dependent variable, analyzed by analysis of covariance (ANCOVA) adjusting for baseline TC and randomization stratification factors (sex, BMI category), comparing adjusted mean 6-month TC across groups; two-sided alpha = 0.05.

Longitudinal outcomes: linear mixed-effects models with fixed effects for group, time (categorical), group × time interaction, baseline outcome value, sex, and BMI category, plus a participant-specific random intercept; adjusted means and 95% confidence intervals will be reported at each time point.

Pairwise comparisons: three prespecified contrasts (HIIT vs control, MICT vs control, HIIT vs MICT), reported as adjusted mean differences with 95% confidence intervals.

Multiple-comparison correction: Holm-adjusted P values for pairwise comparisons; false-discovery-rate control within secondary outcome families.

4. Missing data and effect estimation: all randomized participants remain in the primary analysis; missing values will be addressed by multiple imputation by chained equations (MICE), estimates pooled by Rubin's rules, with complete-case and per-protocol analyses and a sensitivity analysis under departures from the missing-at-random assumption; adjusted mean differences and 95% confidence intervals will be emphasized.

5. Correlation analysis: multivariable linear regression with change from baseline in ET-1 or NO as the dependent variable, and changes in individual glycolipid markers (TC, TG, HDL-C, LDL-C, FPG, FINS, HOMA-IR) entered separately as predictors (to avoid multicollinearity), adjusting for group, baseline endothelial outcome, sex, and BMI category; standardized regression coefficients, 95% CIs, and P values will be reported.

6. Subgroup and sensitivity analyses: exploratory sex-stratified subgroup analysis; per-protocol analysis as a sensitivity check; no formal efficacy claim will be based solely on subgroup or per-protocol analyses.

7. Software: SPSS 25.0 and R. Two-sided tests; $P < 0.05$ considered statistically significant.

Part 6. Timeline and Budget

1. Detailed timeline (Gantt chart)

Phase	M1-2	M3	M4-9	M10	M11	M12
Preparation (protocol, ethics, preparation)	████	███				
Implementation (recruitment, intervention, follow-up)			██████			
Analysis (data cleaning, statistics)				████	████	
Dissemination (manuscript, submission)						████

2. Detailed budget

Category	Main use	Amount (RMB)
Reagents and consumables	Glucose, insulin, lipid, ET-1 assays; blood-collection supplies	26,000
Equipment and venue	Basic heart-rate monitoring devices; pool access	6,500
Personnel	Research assistant stipend	8,000
Publication and dissemination	Article processing charges	11,500
Total		50,000

Part 7. Ethics, Risk Control, and Contingency Plan

1. Ethical principles

- This protocol has been reviewed and approved by the Scientific Research Ethics Review Committee of Shanxi University (approval no. SXULL2026081).

- The study will be conducted in strict accordance with the Declaration of Helsinki, ensuring informed consent, voluntary participation, freedom to withdraw, confidentiality, non-maleficence, and maximization of benefit.

2. Risk identification and prevention

Exercise-related risks (muscle strain, cardiovascular events): prevent through rigorous screening to exclude high-risk individuals; warm-up before and cool-down after each session; continuous coach supervision with an AED on site; progressive intensity.

Blood-collection risks (pain, infection, vasovagal reaction): performed by a licensed nurse under sterile conditions; candy and a supine blood-collection area prepared for vasovagal reactions.

Privacy breach risks: anonymized data; encrypted storage; access restricted to study team members.

3. Adverse event management and contingency plan

Definition and recording: any unexpected medical event related or unrelated to the intervention will be recorded and reported to the principal investigator within 24 hours.

Management: minor events (e.g., mild muscle soreness) will be managed on site with observation; serious events (e.g., exercise injury, syncope) will immediately stop the intervention, transfer to the university hospital or call 120, and notify family; the ethics committee will be notified within 24 hours.

Withdrawal criteria: occurrence of a serious adverse event; participant request to withdraw; or the investigator judges continued intervention to carry excessive risk.

Part 8. Expected Outcomes and Translation

1. Academic outputs: 1-2 papers in SCI/SSCI-indexed journals; training of 1-2 graduate students.

2. Policy and practice value: an evidence-based Swimming Exercise Intervention Operating Manual for obese college students, for use by university physical education departments and student health centers.

3. Social benefit: raise college students' awareness of scientific exercise for metabolic health, providing a feasible strategy to curb rising obesity rates.

4. Long-term development: accumulate data and experience for the team's future research on the molecular mechanisms of different exercise modalities on metabolic health.
