# Supplementary material for: High-intensity interval versus moderate-intensity continuous freestyle swimming in obese college students: A randomized controlled study protocol examining glycolipid and vascular endothelial responses
Source: PLoS One. 2026 Oct 30;21(10):e0360473. doi: 10.1371/journal.pone.0360473 (PMC13722204; doi:10.1371/journal.pone.0360473)
Supplement: Supplementary file 3 [file pone.0360473.s004.docx]

| **Journal** | PLOS ONE |
| --- | --- |
| **Manuscript ID** | PONE-D-26-18550 |
| **Manuscript title** | High-intensity interval versus moderate-intensity continuous freestyle swimming in obese college students: a randomized controlled study protocol examining glycolipid and vascular endothelial responses |

Dear Dr. Maryam Koushkie Jahromi and Reviewers,

We sincerely thank the Academic Editor and both reviewers for their careful and constructive evaluation of our manuscript. We have revised the protocol extensively to improve methodological transparency, statistical prespecification, privacy protection, and compliance with PLOS ONE and SPIRIT 2025 requirements. Our point-by-point responses are provided below. All locations refer to the current marked-up revision. Line references are limited to the numbered main text (lines 1-340); references and supporting information are identified by section or reference number so that automatically continued line numbers are not mistaken for main-text locations.

# **A. Journal Requirements**

*Responses to the ten additional requirements listed by the PLOS ONE editorial office.*

## **A1. PLOS ONE style and file naming**

**Comment:** *Please ensure that the manuscript meets PLOS ONE style requirements, including file naming.*

**Response:** We revised the manuscript structure, figure citations, supporting-information captions, reference presentation, and submission-file labels to conform to PLOS ONE requirements. The marked-up manuscript is named and prepared as a separate revision file; a clean manuscript and separate figure files will accompany the final resubmission.

**Changes in the revised manuscript:** *Global formatting and file-organization revisions throughout the manuscript.*

## **A2. Corresponding-author ORCID validation**

**Comment:** *PLOS requires a validated ORCID iD for the corresponding author in Editorial Manager.*

**Response:** We added the ORCID iDs supplied by all five authors: Mei Yang, 0009-0003-7327-7887; Jingjing Wang, 0009-0005-9416-697X; Xiaoyun Su, 0000-0002-8369-2190; Huan Xiang, 0009-0007-5211-0104; and Yuting Hou, 0009-0006-1612-5985. The corresponding author will also complete the journal's required validation through Editorial Manager before resubmission.

**Changes in the revised manuscript:** *Author information, lines 11-12.*

## **A3. Funding-information mismatch**

**Comment:** *The grant information in the Funding Information and Financial Disclosure sections does not match.*

**Response:** We apologize for the inconsistency. We have checked and modified the fund information.

## **A4. Role of the funder**

**Comment:** *Please state the role of the funders; if they had no role, use the journal’s specified statement and include it in the cover letter.*

**Response:** This article has No individual self-help. The funding source of this article is the Shanxi Province Postgraduate Education Reform Research Project (Grant No. 2021YJJG001). The project leader is Yang Mei.

## **A5. Removal of funding text from the manuscript**

**STATUS: ADDRESSED**

**Comment:** *Funding information should not appear in the manuscript and should be removed from all manuscript sections.*

**Response:** We removed the funding section and all funding-related text from the revised manuscript. Funding information will be provided only through the journal’s online Funding Statement and the cover letter, as requested.

**Changes in the revised manuscript:** *Funding row/section removed; no funding statement remains in the manuscript body.*

## **A6. Third-party data contact**

**Comment:** *The Data Availability Statement is missing the name and contact details of the third party from whom restricted data may be requested.*

**Response:** Response: We have revised the data availability statement, appointing the Research Ethics Review Committee of Shanxi University as the institution responsible for restricting data access procedures. However, since the Research Ethics Review Committee of Shanxi University does not directly accept direct access and data requests from the public, we will have the corresponding author review the reasons for the data requirements and initiate the application or directly provide the available data.

**Changes in the revised manuscript:** *Data Availability Statement, lines 327-332.*

## **A7. Placement of the ethics statement**

**STATUS: ADDRESSED**

**Comment:** *The ethics statement should appear only in the Methods section.*

**Response:** We consolidated the ethics approval, trial registration, informed-consent procedure, and Declaration of Helsinki statement under ‘Study design and ethics’ in the Materials and methods section and removed duplicate ethics text elsewhere.

**Changes in the revised manuscript:** *Materials and methods, ‘Study design and ethics,’ lines 81-88.*

## **A8. Supporting Information captions**

**Comment:** *Please include captions for all Supporting Information files at the end of the manuscript and update in-text citations accordingly.*

**Response:** We retained a Supporting information section at the end of the manuscript with a caption for the completed SPIRIT 2025 checklist. The previously submitted preliminary-data file is no longer cited.

**Changes in the revised manuscript:** *Supporting information section (after Reference 25).*

## **A9. English-language underlying dataset**

**STATUS: ADDRESSED**

**Comment:** *The submitted dataset contains text or data that is not in English; an English-language version is required.*

**Response:** The non-English preliminary-data file has been removed from the revision because it is no longer used or cited. No trial dataset was generated for preparation of this protocol. The sample-size calculation is now based on published studies, which are fully cited in the manuscript [23,24], and no S1 Data file is required for that calculation.

**Changes in the revised manuscript:** *Participants and sample size, lines 118-129; Data Availability Statement, lines 327-332; References 23-24; Supporting information section.*

## **A10. Evaluation of suggested references**

**STATUS: ADDRESSED**

**Comment:** *Authors should evaluate reviewer-suggested literature and are not required to cite irrelevant works unless instructed by the editor.*

**Response:** We evaluated the literature needs raised by the reviewers and added or replaced only sources that directly support the revised statements. In particular, we added a recent college-student prevalence source, replaced the conference proceeding previously cited as Reference 13 with a peer-reviewed meta-analysis of aquatic endurance exercise, cited SPIRIT 2025, added a source addressing metabolically healthy obesity, and added two published exercise trials plus the G*Power source for the sample-size rationale.

**Changes in the revised manuscript:** *Introduction, lines 38-58; Participants and sample size, lines 118-129; References 2, 13, 17, and 22-25.*

# **B. Additional Academic Editor Comments**

*Responses to the six mandatory editorial issues highlighted in the Major Revision decision.*

## **B1. Ethical and privacy compliance in Figure 3**

**STATUS: ADDRESSED**

**Comment:** *Figure 3 contains identifiable participant information. Permanently redact all PHI/PII or replace the image with a generic DEXA schematic.*

**Response:** We fully agree. The original DEXA report has been removed and replaced with a generic, de-identified schematic showing the assessment workflow and prespecified body-composition outputs. The new figure contains no participant identifiers, dates, demographic fields, or individual clinical values.

**Changes in the revised manuscript:** *Figure 3 and caption, lines 131-133.*

## **B2. SPIRIT reporting guideline and prospective flow diagram**

**Comment:** *Use SPIRIT rather than CONSORT, update the text, populate or reformat Figure 2 prospectively, correct ‘conteol,’ and upload a completed SPIRIT checklist.*

**Response:** We replaced CONSORT with SPIRIT 2025 in the manuscript. Figure 2 has been rebuilt as a prospective participant-flow diagram with a target randomized sample of 108 and planned allocation of 36 participants to each arm; screening, exclusion, follow-up, and analysis fields are explicitly identified as values to be reported after trial completion. The typographical error ‘conteol’ has been corrected to ‘control.’ The manuscript also includes a caption for the SPIRIT 2025 checklist.

**Changes in the revised manuscript:** *Participant timeline, lines 72-76; Study design and ethics, lines 81-88; Figure 2 and caption, lines 89-92; Supporting information section.*

## **B3. Statistical power and outcome hierarchy**

**STATUS: ADDRESSED**

**Comment:** *Define one primary outcome or co-primary outcomes, classify the remainder, discuss limited power for secondary/exploratory outcomes, and provide all exact G*Power inputs and the source of preliminary data.*

**Response:** We now define 6-month total cholesterol (TC), adjusted for baseline TC, as the single primary outcome. The sample-size calculation no longer relies on unpublished preliminary data. It is anchored to published aquatic-exercise and obesity exercise trials [23,24]. Tang et al. reported TC changes from 5.36 ± 0.84 to 4.96 ± 1.00 mmol/L after aquatic HIIT and from 5.06 ± 0.81 to 5.03 ± 0.40 mmol/L after aquatic MICT; Saeidi et al. reported an effect size of 0.90 for the TC group-by-time comparison. Because those trials differ from the present design, we used a smaller, conservative moderate effect, Cohen's f = 0.325 (eta-squared = 0.096). The a priori G*Power 3.1.9.7 specification was F tests; ANOVA: fixed effects, omnibus, one-way; three groups; alpha = 0.05; power = 0.80. It yielded 95 participants, rounded to 96 for equal group sizes. Inflating 96 by 10% gives 106.7, rounded to 108 (36 per group).

**Changes in the revised manuscript:** *Participants and sample size, lines 118-129; Outcome measures, lines 159-194; Statistical analysis, lines 217-255; Limitations, lines 298-304; References 23-25.*

## **B4. Analytical plan, missing data, metabolic-endothelial relationships, and stratification**

**Comment:** *Prespecify ANCOVA; describe missing-data and dropout handling; operationalize metabolic-vascular coupling; and justify the randomization strata.*

**Response:** We extensively revised the statistical analysis plan. The primary analysis is now a prespecified intention-to-treat ANCOVA of 6-month TC, adjusted for baseline TC, sex, and BMI category, irrespective of observed baseline balance. Repeated secondary outcomes will be analyzed with linear mixed-effects models. Missing primary outcomes or covariates will be addressed using multiple imputation by chained equations, with complete-case, per-protocol, and departures-from-missing-at-random sensitivity analyses. Additional multivariable linear regression models will examine relationships between changes in individual glycolipid markers and concurrent changes in ET-1 and NO at 2, 4, and 6 months. Stratification has been revised to sex and baseline BMI category, with a physiological and methodological rationale.

**Changes in the revised manuscript:** *Randomization and blinding, lines 135-142; Statistical analysis, lines 217-255.*

## **B5. Metabolic phenotype, protocol tense, and sustained-adaptation claims**

**Comment:** *Address MHO/MUO heterogeneity, use future tense consistently, and revise the claim of sustained adaptation because no post-intervention follow-up is planned.*

**Response:** We added baseline metabolic screening and prespecified classification of metabolically healthy obesity (MHO) and metabolically unhealthy obesity (MUO). Phenotype will be included as a covariate and exploratory effect modifier, and phenotype-specific effects are identified as underpowered. We revised the Abstract and planned procedures to future tense. We removed the unsupported claim that MICT will yield post-intervention ‘sustained’ effects and now state explicitly that the protocol ends at 6 months and cannot evaluate durability after exercise cessation.

**Changes in the revised manuscript:** *Abstract, lines 14-35; baseline screening and phenotype classification, lines 109-117; Discussion, lines 263-304.*

## **B6. Methodological detail, references, and Figure 6**

**Comment:** *Expand blood handling and assay methods; replace Reference 13; provide a more appropriate college-student prevalence citation; and update Figure 6 to depict vascular adaptations and remove artifacts.*

**Response:** We added presampling restrictions, collection timing, specimen types, clotting and centrifugation conditions, storage temperature, analytical principles, batch handling, duplicate measurements, quality controls, and a repeat-testing threshold. The manuscript now specifies that the FINS, ET-1, and NO kits will be manufactured by Elabscience Biotechnology Co., Ltd. (Wuhan, China); the actual catalog and production-lot numbers, detection ranges, and intra-/inter-assay coefficients of variation will be recorded during the trial and reported in the final results article. We replaced Reference 13 with a peer-reviewed aquatic-exercise meta-analysis and added a recent national study of Chinese college students. We retained the authors' revised Figure 6, rewrote its caption to explain glucose regulation, lipid transport, blood-flow patterns, and energy balance, and removed the isolated caption artifact.

**Changes in the revised manuscript:** *Introduction, lines 38-58; Blood collection and laboratory assays, lines 180-194; Figure 6 caption, lines 277-282; References 2 and 13.*

# **C. Reviewer 1 — Ching-Ti Liu, Ph.D.**

*The reviewer’s five numbered comments are reproduced and answered individually.*

## **C1. Rationale for randomization stratification**

**Comment:** *Stratified randomization is planned based on height and weight. Please provide reasoning. Why not sex or BMI?*

**Response:** We agree that sex and BMI are more directly interpretable and nonredundant stratification factors than separate height and weight measures. We therefore revised the planned centralized randomization to stratify by sex and baseline BMI category (<30 vs ≥30 kg/m²), using concealed variable block sizes. Sex and BMI were selected because they are expected to influence body composition, metabolic risk, and exercise response; height and weight are recorded but are not used as separate strata because both contribute to BMI.

**Changes in the revised manuscript:** *Randomization and blinding, lines 135-142.*

## **C2. Preliminary data and effect size**

**Comment:** *The sample-size calculation is based on preliminary data, but the preliminary data, reference, and effect size are unclear.*

**Response:** We agree that the original description was insufficient. We removed the statement that the calculation was based on preliminary data and replaced it with a fully traceable literature-based calculation. Published aquatic HIIT/MICT results and an exercise trial in adults with obesity were used to establish a plausible range [23,24], and a smaller conservative omnibus effect (Cohen's f = 0.325; eta-squared = 0.096) was used for planning. The exact G*Power 3.1.9.7 settings were F tests; ANOVA: fixed effects, omnibus, one-way; three groups; alpha = 0.05; and power = 0.80. The computed minimum was 95, rounded to 96 for equal allocation. After 10% attrition, 96/(1-0.10) = 106.7, rounded up to 108 participants (36 per group).

**Changes in the revised manuscript:** *Participants and sample size, lines 118-129; References 23-25.*

## **C3. Missing data and impact on analysis**

**Comment:** *Please explain more explicitly how missing data will be handled and whether it will affect the analysis plan.*

**Response:** We added a prespecified missing-data strategy. All randomized participants will remain in the intention-to-treat analysis. Reasons, timing, and patterns of missingness will be summarized by group. Repeated-outcome models will use maximum likelihood under a missing-at-random assumption. For the primary ANCOVA, missing outcomes or covariates will be multiply imputed using chained equations that include treatment group, baseline and prior outcomes, stratification variables, adherence, and predictors of missingness; estimates will be pooled using Rubin’s rules. Complete-case, per-protocol, and departures-from-missing-at-random sensitivity analyses will assess robustness.

**Changes in the revised manuscript:** *Statistical analysis, lines 237-243.*

## **C4. Prespecification of baseline adjustment**

**Comment:** *Baseline adjustment should be prespecified rather than conditional on observed balance or imbalance.*

**Response:** We agree. Baseline TC and the randomization stratification factors are now included in the primary ANCOVA regardless of observed baseline balance. Baseline significance testing will not be used to select covariates or determine the primary model.

**Changes in the revised manuscript:** *Statistical analysis, lines 222-230.*

## **C5. Single primary outcome and power for other markers**

**Comment:** *Is there a single primary outcome, or will several markers be treated equivalently? If several, the study may be underpowered because sample size appears based on one outcome.*

**Response:** We have clarified that TC concentration at 6 months is the single primary outcome. The primary estimand is the adjusted mean between-group difference under randomized assignment. All other outcomes are explicitly secondary. We state that the study is not powered for individual secondary biomarkers, subgroup comparisons, or the additional regression analyses, and these results will be reported with confidence intervals and appropriate multiplicity procedures.

**Changes in the revised manuscript:** *Participants and sample size, lines 118-129; Outcome measures, lines 159-194; Statistical analysis, lines 217-255; Limitations, lines 298-304.*

# **D. Reviewer 2 — Feroz Akhtar, Ph.D.**

*The reviewer’s major methodological and reporting concerns are answered separately below.*

## **D1. MHO/MUO phenotypic heterogeneity**

**Comment:** *BMI and body-fat criteria treat obesity as uniform and ignore MHO/MUO heterogeneity. Include baseline metabolic screening or justify why the distinction is not relevant; at minimum discuss it as a limitation.*

**Response:** We agree that metabolic phenotype may influence intervention response. We added a structured pre-randomization medical and biochemical screening procedure and prespecified classification using metabolic criteria for triglycerides, HDL-C, blood pressure, fasting glucose, relevant drug treatment, and manifest cardiovascular disease. Both phenotypes are retained in the present draft because the study addresses obesity across the metabolic-risk spectrum; phenotype will be included as a prespecified covariate and exploratory effect modifier. We also added the lack of power for phenotype-specific effects to the limitations.

**Changes in the revised manuscript:** *Participants and sample size, lines 109-117; Limitations, lines 298-304.*

## **D2. Terminology and regression analysis of metabolic-endothelial relationships**

**Comment:** *Define metabolic-vascular coupling as a measurable quantity and specify an analytical framework beyond simple regression association.*

**Response:** Thank you for this important comment. We revised the terminology throughout the title, Abstract, Introduction, hypotheses, outcome definitions, statistical analysis, Discussion, and Figure 6. The revised manuscript now examines relationships between the measured glycolipid markers and ET-1/NO. At each follow-up, change from baseline in ET-1 and change from baseline in NO will be analyzed as separate dependent variables. Changes in TC, TG, HDL-C, LDL-C, FPG, FINS, and HOMA-IR will be entered separately as the main predictor in multivariable linear regression models adjusted for randomized group, the baseline endothelial value, sex, and BMI category. We will report standardized regression coefficients with 95% confidence intervals and apply false-discovery-rate adjustment across the metabolic predictors for each endothelial outcome and time point.

**Changes in the revised manuscript:** *Introduction, lines 59-70; Statistical analysis, lines 244-255; Discussion, lines 283-297.*

## **D3. No post-intervention follow-up and the claim of sustained adaptation**

**Comment:** *The manuscript predicts more stable and sustained MICT adaptations, but the final assessment coincides with trial completion. Why is there no follow-up after 6 months?*

**Response:** We agree that the original wording exceeded the study design. The claim of post-intervention persistence has been removed. The revised Discussion limits inference to trajectories during the 6-month intervention and explicitly states that the absence of a post-intervention follow-up prevents evaluation of durability after exercise cessation. We did not add an unapproved follow-up assessment retrospectively.

**Changes in the revised manuscript:** *Discussion, lines 270-275 and 298-304.*

## **D4. Protocol tense in the Abstract**

**Comment:** *The phrase ‘examined whether’ gives the false impression that the study is complete. Revise the Abstract so verbs reflect a protocol.*

**Response:** We revised the Abstract to future tense throughout. It now states that the trial ‘will compare,’ participants ‘will be randomized,’ outcomes ‘will be assessed,’ and analyses ‘will characterize’ the planned study.

**Changes in the revised manuscript:** *Abstract, lines 14-35.*

## **D5. SPIRIT versus CONSORT and prospective Figure 2**

**Comment:** *CONSORT is for completed trials; cite SPIRIT, populate or reformat Figure 2 prospectively, and correct ‘conteol.’*

**Response:** We replaced CONSORT with SPIRIT 2025 and cited the updated SPIRIT statement in the Methods. Figure 2 has been redrawn as a prospective SPIRIT participant-flow diagram with planned n=108 and n=36 per arm, while actual screening, attrition, and analysis counts are explicitly marked for future reporting. The spelling error has been corrected.

**Changes in the revised manuscript:** *Participant timeline, lines 72-76; Study design and ethics, lines 81-88; Figure 2 and caption, lines 89-92; Reference 17.*

## **D6. De-identification of Figure 3**

**Comment:** *The DEXA report contains identifiable information and must be fully de-identified or replaced with a schematic.*

**Response:** The original participant report has been removed in full. We replaced it with a generic schematic of DEXA positioning, scanning, and prespecified outputs. No individual participant record or identifying field is shown.

**Changes in the revised manuscript:** *Figure 3 and caption, lines 131-133.*

## **D7. Revision of Figure 6**

**Comment:** *Figure 6 should include vascular endothelial adaptations and remove stray artifacts or spots.*

**Response:** We retained the authors' revised Figure 6 exactly as supplied. To make its content explicit, we replaced the brief caption with a point-by-point explanation of pancreatic insulin/glucagon regulation, hepatic glycogen handling, lipid transport and storage, exercise-related increases in blood flow and laminar shear, and whole-body energy balance. We also removed the isolated period below the former caption. The revised caption links these physiological relationships to the metabolic and endothelial outcomes assessed in the trial.

**Changes in the revised manuscript:** *Figure 6 caption, lines 277-282.*

## **D8. College-student obesity prevalence citation**

**Comment:** *The citation at the original lines 39–41 is too broad; cite literature relevant to college students or young adults.*

**Response:** We replaced the broad prevalence support with a recent national cross-sectional study of 291,276 Chinese college students aged 19–22 years, reporting time trends and projected prevalence of overweight and obesity. This source directly matches the study population.

**Changes in the revised manuscript:** *Introduction, lines 38-43; Reference 2.*

## **D9. Replacement of conference-proceeding Reference 13**

**STATUS: ADDRESSED**

**Comment:** *Reference 13 is a conference proceeding and should be replaced with a peer-reviewed journal article.*

**Response:** We replaced the conference proceeding with a peer-reviewed meta-analysis of randomized controlled trials evaluating lipid and lipoprotein responses to regular aquatic endurance exercise.

**Changes in the revised manuscript:** *Introduction, lines 54-58; Reference 13.*

## **D10. Expansion of study design, screening, blood handling, and assays**

**Comment:** *Expand the Materials and Methods to describe study design, medical screening, baseline health assessment, blood handling, and assays for ET-1, NO, and glycolipid outcomes.*

**Response:** We expanded the Methods to specify the three-arm assessor-blinded design, ethics and registration, structured medical and medication review, vital signs, physical examination, exercise-readiness and swimming-ability screening, baseline fasting biochemistry, and physician review. We also added presampling restrictions, collection time, plasma/serum tube selection, clotting, centrifugation, storage, assay principles for FPG, lipids, FINS, ET-1 and NO, the HOMA-IR equation, batch handling, duplicate measurements, internal quality controls, and repeat-testing criteria. The FINS, ET-1, and NO kits are now identified as products of Elabscience Biotechnology Co., Ltd. (Wuhan, China). The actual catalog and production-lot numbers, detection ranges, and intra-/inter-assay coefficients of variation will be recorded during the trial and reported in the final results article.

**Changes in the revised manuscript:** *Study design and ethics, lines 81-88; screening and baseline assessment, lines 109-117; Blood collection and laboratory assays, lines 180-194.*

Sincerely,
Mei Yang, on behalf of all authors
