# Supplementary material for: High-intensity interval versus moderate-intensity continuous freestyle swimming in obese college students: A randomized controlled study protocol examining glycolipid and vascular endothelial responses
Source: PLoS One. 2026 Oct 30;21(10):e0360473. doi: 10.1371/journal.pone.0360473 (PMC13722204; doi:10.1371/journal.pone.0360473)
Supplement: Peer review history

# Peer review: Decision Letter 0

**DOI:** 10.1371/journal.pone.0360473.r001

**Authors:** Maryam Koushkie Jahromi

10 Aug 2026

Dear Dr. Yang,

Thank you for submitting your manuscript to PLOS ONE. After careful consideration, we feel that it has merit but does not fully meet PLOS ONE’s publication criteria as it currently stands. Therefore, we invite you to submit a revised version of the manuscript that addresses the points raised during the review process.

Please submit your revised manuscript by Sep 09 2026 11:59PM. If you will need more time than this to complete your revisions, please reply to this message or contact the journal office at plosone@plos.org. When you're ready to submit your revision, log on to https://www.editorialmanager.com/pone/ and select the 'Submissions Needing Revision' folder to locate your manuscript file.

- A letter that responds to each point raised by the academic editor and reviewer(s). You should upload this letter as a separate file labeled 'Response to Reviewers'.
- A marked-up copy of your manuscript that highlights changes made to the original version. You should upload this as a separate file labeled 'Revised Manuscript with Track Changes'.
- An unmarked version of your revised paper without tracked changes. You should upload this as a separate file labeled 'Manuscript'.

If you would like to make changes to your financial disclosure, please include your updated statement in your cover letter. Guidelines for resubmitting your figure files are available below the reviewer comments at the end of this letter.

If applicable, we recommend that you deposit your laboratory protocols in protocols.io to enhance the reproducibility of your results. Protocols.io assigns your protocol its own identifier (DOI) so that it can be cited independently in the future. For instructions see: https://journals.plos.org/plosone/s/submission-guidelines#loc-laboratory-protocols. Additionally, PLOS ONE offers an option for publishing peer-reviewed Lab Protocol articles, which describe protocols hosted on protocols.io. Read more information on sharing protocols at https://plos.org/protocols?utm_medium=editorial-email&utm_source=authorletters&utm_campaign=protocols.

As the corresponding author, your ORCID iD is verified in the submission system and will appear in the published article. PLOS supports the use of ORCID, and we encourage all coauthors to register for an ORCID iD and use it as well. Please encourage your coauthors to verify their ORCID iD within the submission system before final acceptance, as unverified ORCID iDs will not appear in the published article. *Only*  the individual author can complete the verification step; PLOS staff *cannot*  verify ORCID iDs on behalf of authors.

We look forward to receiving your revised manuscript.

Kind regards,

Maryam Koushkie Jahromi

Academic Editor

PLOS One

Journal Requirements:

When submitting your revision, we need you to address these additional requirements.

1.Please ensure that your manuscript meets PLOS ONE's style requirements, including those for file naming. The PLOS ONE style templates can be found at

https://journals.plos.org/plosone/s/file?id=wjVg/PLOSOne_formatting_sample_main_body.pdf and

https://journals.plos.org/plosone/s/file?id=ba62/PLOSOne_formatting_sample_title_authors_affiliations.pdf

2. PLOS requires an ORCID iD for the corresponding author in Editorial Manager on papers submitted after December 6th, 2016. Please ensure that you have an ORCID iD and that it is validated in Editorial Manager. To do this, go to ‘Update my Information’ (in the upper left-hand corner of the main menu), and click on the Fetch/Validate link next to the ORCID field. This will take you to the ORCID site and allow you to create a new iD or authenticate a pre-existing iD in Editorial Manager.

3. We note that the grant information you provided in the ‘Funding Information’ and ‘Financial Disclosure’ sections do not match.

When you resubmit, please ensure that you provide the correct grant numbers for the awards you received for your study in the ‘Funding Information’ section.

4. Thank you for stating the following financial disclosure:

“2021 Shanxi Province Postgraduate Education Reform Research Project (Grant No. 2021YJJG001)”

Please state what role the funders took in the study.  If the funders had no role, please state: "The funders had no role in study design, data collection and analysis, decision to publish, or preparation of the manuscript."

If this statement is not correct you must amend it as needed.

Please include this amended Role of Funder statement in your cover letter; we will change the online submission form on your behalf.

5. Please note that funding information should not appear in any section or other areas of your manuscript. We will only publish funding information present in the Funding Statement section of the online submission form. Please remove any funding-related text from the manuscript.

6. In the online submission form, you indicated that your data is available only on request from a third party. Please note that your Data Availability Statement is currently missing the name of the third party contact or institution / contact details for the third party, such as an email address or a link to where data requests can be made. Please update your statement with the missing information.

7. Your ethics statement should only appear in the Methods section of your manuscript. If your ethics statement is written in any section besides the Methods, please move it to the Methods section and delete it from any other section. Please ensure that your ethics statement is included in your manuscript, as the ethics statement entered into the online submission form will not be published alongside your manuscript.

8. Please include captions for your Supporting Information files at the end of your manuscript, and update any in-text citations to match accordingly. Please see our Supporting Information guidelines for more information: http://journals.plos.org/plosone/s/supporting-information.

9. Thank you for providing your underlying data as Supporting Information.

We note that the data set contains text or data that is not in English. Please note that PLOS is an English-language publisher, so we require data sets to be provided in English as well. Please upload an English-language version of your data set.

This will also allow us to determine if your data follows PLOS standards per our Data Availability policy here: https://journals.plos.org/plosone/s/data-availability.

10. If the reviewer comments include a recommendation to cite specific previously published works, please review and evaluate these publications to determine whether they are relevant and should be cited. There is no requirement to cite these works unless the editor has indicated otherwise.

Additional Editor Comments:

Dear Dr. Yang and co-authors,

Thank you for submitting your study protocol, "High-intensity interval versus moderate-intensity continuous freestyle swimming in obese college students: a randomized controlled study protocol targeting metabolic-vascular coupling" (PONE-D-26-18550), to PLOS ONE.

Your manuscript has been evaluated by expert reviewers in the fields of exercise physiology and biostatistics. While the reviewers and I find the proposed intervention to be timely, procedurally well-planned, and of significant interest to the field, several critical methodological, statistical, and formatting issues must be addressed before the protocol can be accepted for publication.

Therefore, my decision is Major Revision.

In addition to addressing the specific points raised by the reviewers (appended below), please pay careful attention to the following mandatory editorial requirements:

1. Ethical and Privacy Compliance (Crucial)

Reviewer 2 correctly noted that Figure 3 (the DEXA scan report) contains identifiable participant information, including date of birth, sex, height, weight, ethnicity, and scanning date. PLOS ONE strictly requires that all images of human subjects or medical records be fully de-identified to protect participant privacy. You must either permanently redact all Protected Health Information (PHI) / Personally Identifiable Information (PII) from this image or replace it with a generic schematic illustration of DEXA measurements.

2. Reporting Guidelines and Flow Diagram

As this is a study protocol, the appropriate reporting guideline is SPIRIT, not CONSORT (which is designed for completed trials).

Please update the manuscript text (Line 100) to cite the SPIRIT guidelines.

Ensure your Flow Diagram (Figure 2) is adapted to a prospective SPIRIT flow diagram, showing the planned sample sizes for enrollment, allocation, and analysis.

Please correct the typo "conteol" to "control" in Figure 2.

Ensure your completed SPIRIT checklist is accurately mapped and uploaded as a Supporting Information file.

3. Statistical Power and Outcome Hierarchy

Reviewer 1 raises a critical point regarding your sample size calculation. You state the calculation is based on Total Cholesterol (TC) using "preliminary data," but you have multiple metabolic and vascular outcomes.

You must explicitly define a single primary outcome (or clearly defined co-primary outcomes) and classify the rest as secondary outcomes.

If you are powering the study only for TC, you must discuss the potential lack of statistical power for the other secondary/exploratory outcomes (such as the ET-1/NO axis) in the limitations section.

You must provide the exact parameters used in your G*Power calculation, including the specific effect size, standard deviations, and the source/citation of the "preliminary data."

4. Analytical Plan and Missing Data

Baseline Adjustment: As noted by Reviewer 1, the use of ANCOVA must be prespecified in your statistical analysis plan as a primary analytical approach, rather than being conditional upon finding baseline imbalances.

Missing Data: You must explicitly state how missing data, loss to follow-up, and dropouts will be handled statistically (e.g., Intention-To-Treat analysis, Multiple Imputation, or Linear Mixed-Effects Models).

Metabolic-Vascular Coupling: Reviewer 2 highlights that "coupling" implies a mechanistic or specific statistical relationship, whereas simple regression only tests association. Please explicitly define how "metabolic-vascular coupling" will be operationalized and measured statistically (e.g., cross-lagged panel models, mediation analysis, or specific interaction terms).

Randomization Stratification: Please provide a physiological or methodological rationale for stratifying by height and weight rather than sex or BMI, as suggested by Reviewer 1.

5. Study Design and Terminology

Metabolic Phenotypes: Address Reviewer 2’s concern regarding Metabolically Healthy Obese (MHO) vs. Metabolically Unhealthy Obese (MUO) phenotypes. Either implement a baseline screening step to restrict the inclusion of MUO participants, or explicitly discuss this phenotypic heterogeneity as a limitation that may affect intervention variability.

Tense: Ensure the Abstract and main text consistently use the future tense (e.g., "will examine" instead of "examined"), as this is a protocol for an ongoing study.

Sustained Adaptations: You state MICT may produce "more stable and sustained adaptations over time" (Line 254), but your study ends at 6 months with no detraining/follow-up period. Please revise this claim or clarify that "sustained" refers only to the 6-month intervention period.

6. Methodological Details and References

Expand the "Materials and Methods" section to include detailed protocols for blood sample handling (e.g., centrifugation speeds, storage temperatures) and the specific assay methods (e.g., ELISA, spectrophotometry) for ET-1, NO, and glycolipid markers.

Replace Reference 13 (a conference proceeding) with a peer-reviewed journal article.

Provide a more appropriate citation for lines 39-41 regarding obesity prevalence specifically in college students/young adults, as the current citation (Andersson & Vasan) covers a broader demographic.

Update Figure 6 to include the vascular endothelial adaptations discussed in the text and remove any stray artifacts/spots.

Reviewer Comments

Reviewer 1: Ching-Ti Liu, Ph.D.

Stratified randomization is planned based on height and weight. Please provide reasoning. Why not sex? Or maybe BMI.

Sample size calculation is based on preliminary data. However, it’s unclear what preliminary data is (no reference citation and even no effect size).

Please comment on how the missing data will be handled more explicitly and will this affect the analysis plan.

Baseline adjustment should be prespecified, not condition on balance or unbalance.

Is there any single primary outcome or are there several markers will be considered equivalently? If later, will it lack power with the pre-determined sample size which seems only based on only one single outcome.

Reviewer 2: Feroz Akhtar, Ph.D.

Certain details of the protocol need to be elaborated or clarified to contextualize the hypothesis and signify the findings. By strictly using BMI and body fat as inclusion criteria for the study participants, the authors have treated obesity as a single uniform condition and ignored the prevalence of phenotypic heterogeneity (i.e., metabolically healthy obese [MHO] and metabolically unhealthy obese [MUO]) in the study design. Failure to account for this phenotypic heterogeneity may influence the variability and the effect exerted by HIIT and MICT. The authors should either justify why this distinction is not relevant to their specific hypothesis or, preferably, include a baseline metabolic screening to restrict the inclusion of MUO participants. At a minimum, this limitation must be explicitly discussed in the protocol.

The authors should explain and define "metabolic-vascular coupling” as it is one of the central hypotheses of the study. It has not been defined as a measurable quantity. The regression of metabolic markers on ET-1/NO test association, not coupling in any mechanistic or statistical sense. The authors should specify the exact statistical test or analytical framework they intend to use to capture this coupling.

The authors expect that MICT may produce “more stable and sustained adaptations over time” (line 254), but in the study design, both the final assessment and the trial end at the same time. Why have the authors not included a follow-up assessment after 6 months to address their claim on sustained adaptation?

The sentence "examined whether HIIT and MICT produce different effects..." in the abstract gives the false impression that the study is already completed. Therefore, the authors should revise the abstract to ensure all verbs accurately reflect the protocol nature of the paper.

The authors have cited CONSORT 2025 as a reporting guideline (line 100), but CONSORT is designed for completed trials. The appropriate standard for protocol is SPIRIT, which is submitted as supplementary material but not cited in text. This inconsistency must be corrected. The authors should either populate Figure 2 depicting the flow diagram with planned "n" values or reformat it as a prospective SPIRIT Figure. They should also correct the typo "conteol."

The DEXA scan report presented as Figure 3 is not fully de-identified and includes information on date of birth, sex, height, weight, ethnicity, and scanning date for a specific individual. The authors should provide a fully de-identified image or a schematic illustration of DEXA measurements.

The authors should also update Figure 6, as the vascular endothelial adaptations are discussed and cited in the text but are not depicted in the schematic diagram. Additionally, stray artifacts or spots are still visible in the figure that need to be cleaned.

Authors should cite relevant literature to support their statement at lines 39-41, as the cited paper by Anderson and Vasan evaluates broader age demographics of 18-45 years of age and focuses on cardiovascular risk.

Reference 13 is a conference proceeding; the authors should replace it with a peer-reviewed article.

The authors should expand the Material and Methods sections by adequately describing the study design, medical screening, and baseline health assessment. They should specify blood handling, and detailed assay protocols should be provided for the target markers, including ET-1, NO, and glycolipid metabolism outcome markers.

Instructions for Resubmission

When you are ready to submit your revised manuscript, please include:

A point-by-point Response Letter detailing how and where you have addressed every comment raised by the reviewers and the editor.

A marked-up version of the manuscript (using Track Changes or highlighted text) showing all revisions.

A clean version of the revised manuscript.

Updated Supporting Information files (including the corrected SPIRIT checklist and de-identified figures).

Thank you for your contribution to PLOS ONE. We look forward to receiving your revised manuscript.

Sincerely,

Academic Editor

[Note: HTML markup is below. Please do not edit.]

Reviewer's Responses to Questions

**Comments to the Author**

1. Does the manuscript provide a valid rationale for the proposed study, with clearly identified and justified research questions?

Reviewer #1: Partly

Reviewer #2: Partly

**********

2. Is the protocol technically sound and planned in a manner that will lead to a meaningful outcome and allow testing the stated hypotheses?

Reviewer #1: Yes

Reviewer #2: Partly

**********

3. Is the methodology feasible and described in sufficient detail to allow the work to be replicable?

Reviewer #1: Yes

Reviewer #2: No

**********

4. Have the authors described where all data underlying the findings will be made available when the study is complete?

The PLOS Data policy

Reviewer #1: Yes

Reviewer #2: Yes

**********

5. Is the manuscript presented in an intelligible fashion and written in standard English?

Reviewer #1: Yes

Reviewer #2: Yes

**********

Please use the space provided to explain your answers to the questions above and, if applicable, provide comments about issues authors must address before this protocol can be accepted for publication. You may also include additional comments for the author, including concerns about research or publication ethics.

You may also provide optional suggestions and comments to authors that they might find helpful in planning their study.

Reviewer #1: The study plans to recruit 108 obese college students to evaluate whether there is differential effects of HIIT and MICT freestyle swimming on glycolipid metabolism and vascular endothelial function.

1. Stratified randomization is planned based on height and weight. Please provide reasoning. Why not sex? Or maybe BMI

2. Sample size calculation is based on preliminary data. However, it’s unclear what preliminary data is (no reference citation and even no effect size).

3. Please comment on how the missing data will be handled more explicitly and will this affect the analysis plan.

4. Baseline adjustment should be prespecified, not condition on balance or unbalance.

5. Is there any single primary outcome or are there several markers will be considered equivalently? If later, will it lack power with the pre-determined sample size which seems only based on only one single outcome.

Reviewer #2: The study protocol of Yang et al aims to address an important and understudied area of exercise physiology: whether swimming intensity differentially shapes metabolic and vascular outcomes in young obese individuals. It shows careful procedural planning, which includes G*Power-based sample sizing, three-arm randomization, stratified allocation, assessor blinding, repeated measurement, and a detailed intervention protocol. However, the following concerns should be addressed prior to its publication.

Certain details of the protocol need to be elaborated or clarified to contextualize the hypothesis and signify the findings. By strictly using BMI and body fat as inclusion criteria for the study participants, the authors have treated obesity as a single uniform condition and ignored the prevalence of phenotypic heterogeneity (i.e., metabolically healthy obese [MHO] and metabolically unhealthy obese [MUO]) in the study design. Failure to account for this phenotypic heterogeneity may influence the variability and the effect exerted by HIIT and MICT. The authors should either justify why this distinction is not relevant to their specific hypothesis or, preferably, include a baseline metabolic screening to restrict the inclusion of MUO participants. At a minimum, this limitation must be explicitly discussed in the protocol."

The authors should explain and define "metabolic-vascular coupling” as it is one of the central hypotheses of the study. It has not been defined as a measurable quantity. The regression of metabolic markers on ET-1/NO test association, not coupling in any mechanistic or statistical sense. The authors should specify the exact statistical test or analytical framework they intend to use to capture this coupling.

The authors expect that MICT may produce “more stable and sustained adaptations over time” (line 254), but in the study design, both the final assessment and the trial end at the same time. Why have the authors not included a follow-up assessment after 6 months to address their claim on sustained adaptation?

The sentence "examined whether HIIT and MICT produce different effects..." in the abstract gives the false impression that the study is already completed. Therefore, the authors should revise the abstract to ensure all verbs accurately reflect the protocol nature of the paper.

The authors have cited CONSORT 2025 as a reporting guideline (line 100), but CONSORT is designed for completed trials. The appropriate standard for protocol is SPIRIT, which is submitted as supplementary material but not cited in text. This inconsistency must be corrected. The authors should either populate Figure 2 depicting the flow diagram with planned "n" values or reformat it as a prospective SPIRIT Figure. They should also correct the typo "conteol."

The DEXA scan report presented as Figure 3 is not fully de-identified and includes information on date of birth, sex, height, weight, ethnicity, and scanning date for a specific individual. The authors should provide a fully de-identified image or a schematic illustration of DEXA measurements.

The authors should also update Figure 6, as the vascular endothelial adaptations are discussed and cited in the text but are not depicted in the schematic diagram. Additionally, stray artifacts or spots are still visible in the figure that need to be cleaned. Authors should cite relevant literature to support their statement at lines 39-41, as the cited paper by Anderson and Vasan evaluates broader age demographics of 18-45 years of age and focuses on cardiovascular risk. Reference 13 is a conference proceeding; the authors should replace it with a peer-reviewed article.

The authors should expand the Material and Methods sections by adequately describing the study design, medical screening, and baseline health assessment. They should specify blood handling, and detailed assay protocols should be provided for the target markers, including ET-1, NO, and glycolipid metabolism outcome markers.

**********

what does this mean?). If published, this will include your full peer review and any attached files.

If you choose “no”, your identity will remain anonymous but your review may still be made public.

**Do you want your identity to be public for this peer review?**  For information about this choice, including consent withdrawal, please see our Privacy Policy

Reviewer #1: No

Reviewer #2: No

**********

[NOTE: If reviewer comments were submitted as an attachment file, they will be attached to this email and accessible via the submission site. Please log into your account, locate the manuscript record, and check for the action link "View Attachments". If this link does not appear, there are no attachment files.]

To ensure your figures meet our technical requirements, please review our figure guidelines: https://journals.plos.org/plosone/s/figures

You may also use PLOS’s free figure tool, NAAS, to help you prepare publication quality figures: https://journals.plos.org/plosone/s/figures#loc-tools-for-figure-preparation.

NAAS will assess whether your figures meet our technical requirements by comparing each figure against our figure specifications.

# Author comment: Author response to Decision Letter 1

**DOI:** 10.1371/journal.pone.0360473.r002

19 Aug 2026

Dear Dr. Maryam Koushkie Jahromi and Reviewers,

We sincerely thank the Academic Editor and both reviewers for their careful and constructive evaluation of our manuscript. We have revised the protocol extensively to improve methodological transparency, statistical prespecification, privacy protection, and compliance with PLOS ONE and SPIRIT 2025 requirements. Our point-by-point responses are provided below.

A. Journal Requirements

Responses to the ten additional requirements listed by the PLOS ONE editorial office.

A1. PLOS ONE style and file naming

Comment: Please ensure that the manuscript meets PLOS ONE style requirements, including file naming.

Response: We revised the manuscript structure, figure citations, supporting-information captions, reference presentation, and submission-file labels to conform to PLOS ONE requirements. The marked-up manuscript is named and prepared as a separate revision file; a clean manuscript and separate figure files will accompany the final resubmission.

Changes in the revised manuscript: Global formatting and file-organization revisions throughout the manuscript.

A2. Corresponding-author ORCID validation

Comment: PLOS requires a validated ORCID iD for the corresponding author in Editorial Manager.

Response: We added the ORCID iDs supplied by all five authors: Mei Yang, 0009-0003-7327-7887; Jingjing Wang, 0009-0005-9416-697X; Xiaoyun Su, 0000-0002-8369-2190; Huan Xiang, 0009-0007-5211-0104; and Yuting Hou, 0009-0006-1612-5985. The corresponding author will also complete the journal's required validation through Editorial Manager before resubmission.

Changes in the revised manuscript: Author information, lines 11-12.

A3. Funding-information mismatch

Comment: The grant information in the Funding Information and Financial Disclosure sections does not match.

Response: We apologize for the inconsistency. We have checked and modified the fund information.

A4. Role of the funder

Comment: Please state the role of the funders; if they had no role, use the journal’s specified statement and include it in the cover letter.

Response: This article has No individual self-help. The funding source of this article is the Shanxi Province Postgraduate Education Reform Research Project (Grant No. 2021YJJG001). The project leader is Yang Mei.

A5. Removal of funding text from the manuscript

STATUS: ADDRESSED

Comment: Funding information should not appear in the manuscript and should be removed from all manuscript sections.

Response: We removed the funding section and all funding-related text from the revised manuscript. Funding information will be provided only through the journal’s online Funding Statement and the cover letter, as requested.

Changes in the revised manuscript: Funding row/section removed; no funding statement remains in the manuscript body.

A6. Third-party data contact

Comment: The Data Availability Statement is missing the name and contact details of the third party from whom restricted data may be requested.

Response: Response: We have revised the data availability statement, appointing the Research Ethics Review Committee of Shanxi University as the institution responsible for restricting data access procedures. However, since the Research Ethics Review Committee of Shanxi University does not directly accept direct access and data requests from the public, we will have the corresponding author review the reasons for the data requirements and initiate the application or directly provide the available data.

Changes in the revised manuscript: Data Availability Statement, lines 327-332.

A7. Placement of the ethics statement

STATUS: ADDRESSED

Comment: The ethics statement should appear only in the Methods section.

Response: We consolidated the ethics approval, trial registration, informed-consent procedure, and Declaration of Helsinki statement under ‘Study design and ethics’ in the Materials and methods section and removed duplicate ethics text elsewhere.

Changes in the revised manuscript: Materials and methods, ‘Study design and ethics,’ lines 81-88.

A8. Supporting Information captions

Comment: Please include captions for all Supporting Information files at the end of the manuscript and update in-text citations accordingly.

Response: We retained a Supporting information section at the end of the manuscript with a caption for the completed SPIRIT 2025 checklist. The previously submitted preliminary-data file is no longer cited.

Changes in the revised manuscript: Supporting information section (after Reference 25).

A9. English-language underlying dataset

STATUS: ADDRESSED

Comment: The submitted dataset contains text or data that is not in English; an English-language version is required.

Response: The non-English preliminary-data file has been removed from the revision because it is no longer used or cited. No trial dataset was generated for preparation of this protocol. The sample-size calculation is now based on published studies, which are fully cited in the manuscript [23,24], and no S1 Data file is required for that calculation.

Changes in the revised manuscript: Participants and sample size, lines 118-129; Data Availability Statement, lines 327-332; References 23-24; Supporting information section.

A10. Evaluation of suggested references

STATUS: ADDRESSED

Comment: Authors should evaluate reviewer-suggested literature and are not required to cite irrelevant works unless instructed by the editor.

Response: We evaluated the literature needs raised by the reviewers and added or replaced only sources that directly support the revised statements. In particular, we added a recent college-student prevalence source, replaced the conference proceeding previously cited as Reference 13 with a peer-reviewed meta-analysis of aquatic endurance exercise, cited SPIRIT 2025, added a source addressing metabolically healthy obesity, and added two published exercise trials plus the G*Power source for the sample-size rationale.

Changes in the revised manuscript: Introduction, lines 38-58; Participants and sample size, lines 118-129; References 2, 13, 17, and 22-25.

B. Additional Academic Editor Comments

Responses to the six mandatory editorial issues highlighted in the Major Revision decision.

B1. Ethical and privacy compliance in Figure 3

STATUS: ADDRESSED

Comment: Figure 3 contains identifiable participant information. Permanently redact all PHI/PII or replace the image with a generic DEXA schematic.

Response: We fully agree. The original DEXA report has been removed and replaced with a generic, de-identified schematic showing the assessment workflow and prespecified body-composition outputs. The new figure contains no participant identifiers, dates, demographic fields, or individual clinical values.

Changes in the revised manuscript: Figure 3 and caption, lines 131-133.

B2. SPIRIT reporting guideline and prospective flow diagram

Comment: Use SPIRIT rather than CONSORT, update the text, populate or reformat Figure 2 prospectively, correct ‘conteol,’ and upload a completed SPIRIT checklist.

Response: We replaced CONSORT with SPIRIT 2025 in the manuscript. Figure 2 has been rebuilt as a prospective participant-flow diagram with a target randomized sample of 108 and planned allocation of 36 participants to each arm; screening, exclusion, follow-up, and analysis fields are explicitly identified as values to be reported after trial completion. The typographical error ‘conteol’ has been corrected to ‘control.’ The manuscript also includes a caption for the SPIRIT 2025 checklist.

Changes in the revised manuscript: Participant timeline, lines 72-76; Study design and ethics, lines 81-88; Figure 2 and caption, lines 89-92; Supporting information section.

B3. Statistical power and outcome hierarchy

STATUS: ADDRESSED

Comment: Define one primary outcome or co-primary outcomes, classify the remainder, discuss limited power for secondary/exploratory outcomes, and provide all exact G*Power inputs and the source of preliminary data.

Response: We now define 6-month total cholesterol (TC), adjusted for baseline TC, as the single primary outcome. The sample-size calculation no longer relies on unpublished preliminary data. It is anchored to published aquatic-exercise and obesity exercise trials [23,24]. Tang et al. reported TC changes from 5.36 ± 0.84 to 4.96 ± 1.00 mmol/L after aquatic HIIT and from 5.06 ± 0.81 to 5.03 ± 0.40 mmol/L after aquatic MICT; Saeidi et al. reported an effect size of 0.90 for the TC group-by-time comparison. Because those trials differ from the present design, we used a smaller, conservative moderate effect, Cohen's f = 0.325 (eta-squared = 0.096). The a priori G*Power 3.1.9.7 specification was F tests; ANOVA: fixed effects, omnibus, one-way; three groups; alpha = 0.05; power = 0.80. It yielded 95 participants, rounded to 96 for equal group sizes. Inflating 96 by 10% gives 106.7, rounded to 108 (36 per group).

Changes in the revised manuscript: Participants and sample size, lines 118-129; Outcome measures, lines 159-194; Statistical analysis, lines 217-255; Limitations, lines 298-304; References 23-25.

B4. Analytical plan, missing data, metabolic-endothelial relationships, and stratification

Comment: Prespecify ANCOVA; describe missing-data and dropout handling; operationalize metabolic-vascular coupling; and justify the randomization strata.

Response: We extensively revised the statistical analysis plan. The primary analysis is now a prespecified intention-to-treat ANCOVA of 6-month TC, adjusted for baseline TC, sex, and BMI category, irrespective of observed baseline balance. Repeated secondary outcomes will be analyzed with linear mixed-effects models. Missing primary outcomes or covariates will be addressed using multiple imputation by chained equations, with complete-case, per-protocol, and departures-from-missing-at-random sensitivity analyses. Additional multivariable linear regression models will examine relationships between changes in individual glycolipid markers and concurrent changes in ET-1 and NO at 2, 4, and 6 months. Stratification has been revised to sex and baseline BMI category, with a physiological and methodological rationale.

Changes in the revised manuscript: Randomization and blinding, lines 135-142; Statistical analysis, lines 217-255.

B5. Metabolic phenotype, protocol tense, and sustained-adaptation claims

Comment: Address MHO/MUO heterogeneity, use future tense consistently, and revise the claim of sustained adaptation because no post-intervention follow-up is planned.

Response: We added baseline metabolic screening and prespecified classification of metabolically healthy obesity (MHO) and metabolically unhealthy obesity (MUO). Phenotype will be included as a covariate and exploratory effect modifier, and phenotype-specific effects are identified as underpowered. We revised the Abstract and planned procedures to future tense. We removed the unsupported claim that MICT will yield post-intervention ‘sustained’ effects and now state explicitly that the protocol ends at 6 months and cannot evaluate durability after exercise cessation.

Changes in the revised manuscript: Abstract, lines 14-35; baseline screening and phenotype classification, lines 109-117; Discussion, lines 263-304.

B6. Methodological detail, references, and Figure 6

Comment: Expand blood handling and assay methods; replace Reference 13; provide a more appropriate college-student prevalence citation; and update Figure 6 to depict vascular adaptations and remove artifacts.

Response: We added presampling restrictions, collection timing, specimen types, clotting and centrifugation conditions, storage temperature, analytical principles, batch handling, duplicate measurements, quality controls, and a repeat-testing threshold. The manuscript now specifies that the FINS, ET-1, and NO kits will be manufactured by Elabscience Biotechnology Co., Ltd. (Wuhan, China); the actual catalog and production-lot numbers, detection ranges, and intra-/inter-assay coefficients of variation will be recorded during the trial and reported in the final results article. We replaced Reference 13 with a peer-reviewed aquatic-exercise meta-analysis and added a recent national study of Chinese college students. We retained the authors' revised Figure 6, rewrote its caption to explain glucose regulation, lipid transport, blood-flow patterns, and energy balance, and removed the isolated caption artifact.

Changes in the revised manuscript: Introduction, lines 38-58; Blood collection and laboratory assays, lines 180-194; Figure 6 caption, lines 277-282; References 2 and 13.

C. Reviewer 1 — Ching-Ti Liu, Ph.D.

The reviewer’s five numbered comments are reproduced and answered individually.

C1. Rationale for randomization stratification

Comment: Stratified randomization is planned based on height and weight. Please provide reasoning. Why not sex or BMI?

Response: We agree that sex and BMI are more directly interpretable and nonredundant stratification factors than separate height and weight measures. We therefore revised the planned centralized randomization to stratify by sex and baseline BMI category (<30 vs ≥30 kg/m²), using concealed variable block sizes. Sex and BMI were selected because they are expected to influence body composition, metabolic risk, and exercise response; height and weight are recorded but are not used as separate strata because both contribute to BMI.

Changes in the revised manuscript: Randomization and blinding, lines 135-142.

C2. Preliminary data and effect size

Comment: The sample-size calculation is based on preliminary data, but the preliminary data, reference, and effect size are unclear.

Response: We agree that the original description was insufficient. We removed the statement that the calculation was based on preliminary data and replaced it with a fully traceable literature-based calculation. Published aquatic HIIT/MICT results and an exercise trial in adults with obesity were used to establish a plausible range [23,24], and a smaller conservative omnibus effect (Cohen's f = 0.325; eta-squared = 0.096) was used for planning. The exact G*Power 3.1.9.7 settings were F tests; ANOVA: fixed effects, omnibus, one-way; three groups; alpha = 0.05; and power = 0.80. The computed minimum was 95, rounded to 96 for equal allocation. After 10% attrition, 96/(1-0.10) = 106.7, rounded up to 108 participants (36 per group).

Changes in the revised manuscript: Participants and sample size, lines 118-129; References 23-25.

C3. Missing data and impact on analysis

Comment: Please explain more explicitly how missing data will be handled and whether it will affect the analysis plan.

Response: We added a prespecified missing-data strategy. All randomized participants will remain in the intention-to-treat analysis. Reasons, timing, and patterns of missingness will be summarized by group. Repeated-outcome models will use maximum likelihood under a missing-at-random assumption. For the primary ANCOVA, missing outcomes or covariates will be multiply imputed using chained equations that include treatment group, baseline and prior outcomes, stratification variables, adherence, and predictors of missingness; estimates will be pooled using Rubin’s rules. Complete-case, per-protocol, and departures-from-missing-at-random sensitivity analyses will assess robustness.

Changes in the revised manuscript: Statistical analysis, lines 237-243.

C4. Prespecification of baseline adjustment

Comment: Baseline adjustment should be prespecified rather than conditional on observed balance or imbalance.

Response: We agree. Baseline TC and the randomization stratification factors are now included in the primary ANCOVA regardless of observed baseline balance. Baseline significance testing will not be used to select covariates or determine the primary model.

Changes in the revised manuscript: Statistical analysis, lines 222-230.

C5. Single primary outcome and power for other markers

Comment: Is there a single primary outcome, or will several markers be treated equivalently? If several, the study may be underpowered because sample size appears based on one outcome.

Response: We have clarified that TC concentration at 6 months is the single primary outcome. The primary estimand is the adjusted mean between-group difference under randomized assignment. All other outcomes are explicitly secondary. We state that the study is not powered for individual secondary biomarkers, subgroup comparisons, or the additional regression analyses, and these results will be reported with confidence intervals and appropriate multiplicity procedures.

Changes in the revised manuscript: Participants and sample size, lines 118-129; Outcome measures, lines 159-194; Statistical analysis, lines 217-255; Limitations, lines 298-304.

D. Reviewer 2 — Feroz Akhtar, Ph.D.

The reviewer’s major methodological and reporting concerns are answered separately below.

D1. MHO/MUO phenotypic heterogeneity

Comment: BMI and body-fat criteria treat obesity as uniform and ignore MHO/MUO heterogeneity. Include baseline metabolic screening or justify why the distinction is not relevant; at minimum discuss it as a limitation.

Response: We agree that metabolic phenotype may influence intervention response. We added a structured pre-randomization medical and biochemical screening procedure and prespecified classification using metabolic criteria for triglycerides, HDL-C, blood pressure, fasting glucose, relevant drug treatment, and manifest cardiovascular disease. Both phenotypes are retained in the present draft because the study addresses obesity across the metabolic-risk spectrum; phenotype will be included as a prespecified covariate and exploratory effect modifier. We also added the lack of power for phenotype-specific effects to the limitations.

Changes in the revised manuscript: Participants and sample size, lines 109-117; Limitations, lines 298-304.

D2. Terminology and regression analysis of metabolic-endothelial relationships

Comment: Define metabolic-vascular coupling as a measurable quantity and specify an analytical framework beyond simple regression association.

Response: Thank you for this important comment. We revised the terminology throughout the title, Abstract, Introduction, hypotheses, outcome definitions, statistical analysis, Discussion, and Figure 6. The revised manuscript now examines relationships between the measured glycolipid markers and ET-1/NO. At each follow-up, change from baseline in ET-1 and change from baseline in NO will be analyzed as separate dependent variables. Changes in TC, TG, HDL-C, LDL-C, FPG, FINS, and HOMA-IR will be entered separately as the main predictor in multivariable linear regression models adjusted for randomized group, the baseline endothelial value, sex, and BMI category. We will report standardized regression coefficients with 95% confidence intervals and apply false-discovery-rate adjustment across the metabolic predictors for each endothelial outcome and time point.

Changes in the revised manuscript: Introduction, lines 59-70; Statistical analysis, lines 244-255; Discussion, lines 283-297.

D3. No post-intervention follow-up and the claim of sustained adaptation

Comment: The manuscript predicts more stable and sustained MICT adaptations, but the final assessment coincides with trial completion. Why is there no follow-up after 6 months?

Response: We agree that the original wording exceeded the study design. The claim of post-intervention persistence has been removed. The revised Discussion limits inference to trajectories during the 6-month intervention and explicitly states that the absence of a post-intervention follow-up prevents evaluation of durability after exercise cessation. We did not add an unapproved follow-up assessment retrospectively.

Changes in the revised manuscript: Discussion, lines 270-275 and 298-304.

D4. Protocol tense in the Abstract

Comment: The phrase ‘examined whether’ gives the false impression that the study is complete. Revise the Abstract so verbs reflect a protocol.

Response: We revised the Abstract to future tense throughout. It now states that the trial ‘will compare,’ participants ‘will be randomized,’ outcomes ‘will be assessed,’ and analyses ‘will characterize’ the planned study.

Changes in the revised manuscript: Abstract, lines 14-35.

D5. SPIRIT versus CONSORT and prospective Figure 2

Comment: CONSORT is for completed trials; cite SPIRIT, populate or reformat Figure 2 prospectively, and correct ‘conteol.’

Response: We replaced CONSORT with SPIRIT 2025 and cited the updated SPIRIT statement in the Methods. Figure 2 has been redrawn as a prospective SPIRIT participant-flow diagram with planned n=108 and n=36 per arm, while actual screening, attrition, and analysis counts are explicitly marked for future reporting. The spelling error has been corrected.

Changes in the revised manuscript: Participant timeline, lines 72-76; Study design and ethics, lines 81-88; Figure 2 and caption, lines 89-92; Reference 17.

D6. De-identification of Figure 3

Comment: The DEXA report contains identifiable information and must be fully de-identified or replaced with a schematic.

Response: The original participant report has been removed in full. We replaced it with a generic schematic of DEXA positioning, scanning, and prespecified outputs. No individual participant record or identifying field is shown.

Changes in the revised manuscript: Figure 3 and caption, lines 131-133.

D7. Revision of Figure 6

Comment: Figure 6 should include vascular endothelial adaptations and remove stray artifacts or spots.

Response: We retained the authors' revised Figure 6 exactly as supplied. To make its content explicit, we replaced the brief caption with a point-by-point explanation of pancreatic insulin/glucagon regulation, hepatic glycogen handling, lipid transport and storage, exercise-related increases in blood flow and laminar shear, and whole-body energy balance. We also removed the isolated period below the former caption. The revised caption links these physiological relationships to the metabolic and endothelial outcomes assessed in the trial.

Changes in the revised manuscript: Figure 6 caption, lines 277-282.

D8. College-student obesity prevalence citation

Comment: The citation at the original lines 39–41 is too broad; cite literature relevant to college students or young adults.

Response: We replaced the broad prevalence support with a recent national cross-sectional study of 291,276 Chinese college students aged 19–22 years, reporting time trends and projected prevalence of overweight and obesity. This source directly matches the study population.

Changes in the revised manuscript: Introduction, lines 38-43; Reference 2.

D9. Replacement of conference-proceeding Reference 13

STATUS: ADDRESSED

Comment: Reference 13 is a conference proceeding and should be replaced with a peer-reviewed journal article.

Response: We replaced the conference proceeding with a peer-reviewed meta-analysis of randomized controlled trials evaluating lipid and lipoprotein responses to regular aquatic endurance exercise.

Changes in the revised manuscript: Introduction, lines 54-58; Reference 13.

D10. Expansion of study design, screening, blood handling, and assays

Comment: Expand the Materials and Methods to describe study design, medical screening, baseline health assessment, blood handling, and assays for ET-1, NO, and glycolipid outcomes.

Response: We expanded the Methods to specify the three-arm assessor-blinded design, ethics and registration, structured medical and medication review, vital signs, physical examination, exercise-readiness and swimming-ability screening, baseline fasting biochemistry, and physician review. We also added presampling restrictions, collection time, plasma/serum tube selection, clotting, centrifugation, storage, assay principles for FPG, lipids, FINS, ET-1 and NO, the HOMA-IR equation, batch handling, duplicate measurements, internal quality controls, and repeat-testing criteria. The FINS, ET-1, and NO kits are now identified as products of Elabscience Biotechnology Co., Ltd. (Wuhan, China). The actual catalog and production-lot numbers, detection ranges, and intra-/inter-assay coefficients of variation will be recorded during the trial and reported in the final results article.

Changes in the revised manuscript: Study design and ethics, lines 81-88; screening and baseline assessment, lines 109-117; Blood collection and laboratory assays, lines 180-194.

Sincerely,

Mei Yang, on behalf of all authors

Attachment Submitted filename: PONE question list.docx

# Editor report: Decision Letter 1

**DOI:** 10.1371/journal.pone.0360473.r003

**Authors:** Maryam Koushkie Jahromi

15 Sep 2026

High-intensity interval versus moderate-intensity continuous freestyle swimming in obese college students: a randomized controlled study protocol targeting metabolic-vascular coupling

PONE-D-26-18550R1

Dear Dr. Yang,

We’re pleased to inform you that your manuscript has been judged scientifically suitable for publication and will be formally accepted for publication once it meets all outstanding technical requirements.

Within one week, you’ll receive an e-mail detailing the required amendments. When these have been addressed, you’ll receive a formal acceptance letter and your manuscript will be scheduled for publication.

An invoice will be generated when your article is formally accepted. Please note, if your institution has a publishing partnership with PLOS and your article meets the relevant criteria, all or part of your publication costs will be covered. Please make sure your user information is up-to-date by logging into Editorial Manager at Editorial Manager® and clicking the ‘Update My Information' link at the top of the page. For questions related to billing, please contact billing support.

If your institution or institutions have a press office, please notify them about your upcoming paper to help maximize its impact. If they’ll be preparing press materials, please inform our press team as soon as possible -- no later than 48 hours after receiving the formal acceptance. Your manuscript will remain under strict press embargo until 2 pm Eastern Time on the date of publication. For more information, please contact onepress@plos.org.

Kind regards,

Maryam Koushkie Jahromi

Academic Editor

PLOS One

Additional Editor Comments (optional):

Dear Dr. Yang and co-authors,

Thank you for submitting your revised manuscript to PLOS ONE. I am pleased to inform you that, following a thorough evaluation of your revised protocol and your detailed point-by-point responses, your manuscript has been accepted for publication.

The authors have done an outstanding job addressing the extensive methodological, statistical, and ethical concerns raised during the peer review process. Specifically, the editorial team and reviewers noted the following significant improvements:

Ethical Compliance: The prompt replacement of the identifiable DEXA scan (Figure 3) with a de-identified schematic successfully resolved the critical privacy concern.

Methodological Rigor: The revision of the randomization stratification factors (to sex and BMI), the explicit definition of a single primary outcome (6-month TC), and the transparent, literature-anchored sample size calculation have greatly strengthened the protocol's scientific validity.

Statistical Transparency: The prespecification of the ANCOVA model (regardless of baseline balance), the robust plan for handling missing data via multiple imputation, and the clear operationalization of the metabolic-endothelial relationship analyses meet the highest standards for clinical trial protocols.

Reporting Guidelines: The seamless transition from CONSORT to the SPIRIT 2025 guidelines, including the prospective flow diagram and checklist, ensures the manuscript aligns perfectly with PLOS ONE requirements for study protocols.

Next Steps in Production:

Your manuscript will now be transferred to our production team. Within the next few days, you will receive an email from our production department with instructions on how to review and approve your typeset proofs.

Please note: As PLOS ONE does not provide copyediting services, it is crucial that you carefully check the proofs for any typographical errors, formatting issues, or missing information before final publication.

Please ensure that your completed SPIRIT 2025 checklist and the updated, de-identified figures are correctly uploaded as Supporting Information files in the final production portal.

Thank you for your rigorous approach to this revision and for choosing PLOS ONE to publish your important work on exercise physiology and metabolic health in young adults. We look forward to publishing your protocol.

Sincerely,

Academic Editor, PLOS ONE

Reviewers' comments:

# Editor report: Acceptance letter

**DOI:** 10.1371/journal.pone.0360473.r004

**Authors:** Maryam Koushkie Jahromi

PONE-D-26-18550R1

PLOS One

Dear Dr. Yang,

I'm pleased to inform you that your manuscript has been deemed suitable for publication in PLOS One. Congratulations! Your manuscript is now being handed over to our production team.

At this stage, our production department will prepare your paper for publication. This includes ensuring the following:

* All references, tables, and figures are properly cited

* All relevant supporting information is included in the manuscript submission,

* There are no issues that prevent the paper from being properly typeset

You will receive further instructions from the production team, including instructions on how to review your proof when it is ready. Please keep in mind that we are working through a large volume of accepted articles, so please give us a few days to review your paper and let you know the next and final steps.

Lastly, if your institution or institutions have a press office, please let them know about your upcoming paper now to help maximize its impact. If they'll be preparing press materials, please inform our press team within the next 48 hours. Your manuscript will remain under strict press embargo until 2 pm Eastern Time on the date of publication. For more information, please contact onepress@plos.org.

You will receive an invoice from PLOS for your publication fee after your manuscript has reached the completed accept phase. If you receive an email requesting payment before acceptance or for any other service, this may be a phishing scheme. Learn how to identify phishing emails and protect your accounts at https://explore.plos.org/phishing.

If we can help with anything else, please email us at customercare@plos.org.

Thank you for submitting your work to PLOS One and supporting open access.

Kind regards,

PLOS One Editorial Office Staff

on behalf of

Dr. Maryam Koushkie Jahromi

Academic Editor

PLOS One
